# Scaling Up Is Not the Same but Bigger: Overcoming (Some) Limitations in Enzymatic Decarboxylations in Rotating Bed Reactors in Deep Eutectic Solvents

**DOI:** 10.1021/acs.oprd.6c00074

**Published:** 2026-05-28

**Authors:** Sonja Vaupel, Lars-Erik Meyer, Pablo Domínguez de María, Selin Kara

**Affiliations:** † Institute of Technical Chemistry, Leibniz University Hannover, Callinstr. 5, 30167 Hannover, Germany; ‡ Sustainable Momentum, SL. Av. Ansite 3, 4-6, 35011 Las Palmas de Gran Canaria, Canary Islands, Spain; § Biocatalysis and Bioprocessing Group, Department of Biological and Chemical Engineering, 1006 Aarhus University, Gustav Wieds Vej 10D, 8000 Aarhus C, Denmark

**Keywords:** phenolic acid decarboxylase, deep eutectic solvents, process intensification, rotating bed reactor, downstream processing

## Abstract

Deep eutectic solvents (DESs) can be designed to be enzyme-compatible and substrate solubilizers. Most biocatalytic reactions in DESs are conducted at small scale, and assessing how to scale up DES-based enzymatic reactions is necessary to assess their feasibility and associated challenges. This work studies the viability of using DESs with a phenolic acid decarboxylase (PAD) to synthesize 4-vinylguaiacol (4-VG) from ferulic acid. Upon PAD immobilization, the reaction is performed in a betaine–glycerol DES (1:2) with 20 vol % buffer in a rotating bed reactor (RBR) at the 120 mL scale. Full conversion of 300 mM ferulic acid (∼58 g/L) is achieved within 18 h at the 10 mL scale. However, challenges a substrate loadings are intended (1 M ferulic acid, ∼200 g/L) in the RBR at 120 mL, where ∼60% conversion is obtained, with a productivity of ∼10 g product/L/h. The formation of CO_2_ during the decarboxylation adds complexity at high substrate loadings, as part of the CO_2_ remains in the DESs and may change the physical–chemical properties. The produced 4-VG can be extracted with heptane (∼29% high-purity, one single extraction step). Overall, results demonstrate the feasibility of the DES/biocatalyst in RBRs for industrial applications, although they also reflect unexpected challenges (e.g., CO_2_ accumulation, low conversions). Research is needed to understand the pre-scale-up of DES-biocatalytic systems, as well as in the identification of biogenic alternatives for liquid–liquid separations.

## Graphical abstract

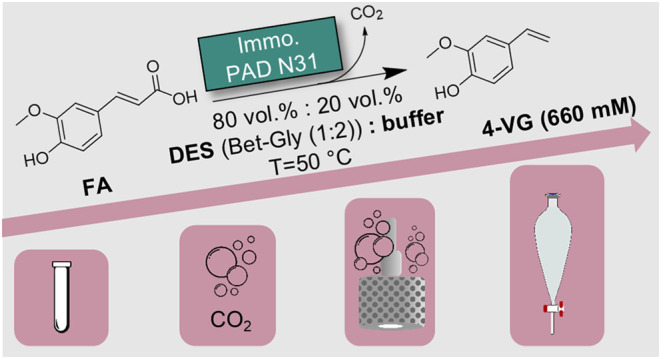

## Introduction

The increasing demand for biobased building blocks for polymers can be attributed to the growing need for sustainable, environmentally friendly materials to advance the circular economy. While fossil-based polymers deplete nonrenewable resources, biobased alternatives offer a renewable and potentially less harmful solution.^1,2^ Transitioning to these biobased components may also be aligned with the promotion of (industrial) reuse and recycling of materials.^3^ Hereby, renewable lignin-originated styrene monomers can play a key role as versatile precursors for synthetic polymers, enabling diverse modifications to tailor their properties. Applications include polymer blends, resins, copolymers, self-healing materials, adhesives, and hydrogels.^4–8^ One promising approach for the valorization of these lignin derivatives is the enzymatic decarboxylation of hydroxycinnamic acids, such as caffeic acid, *p*-coumaric acid, and ferulic acid, to afford hydroxystyrenes that can be used for polymer production, catalyzed by phenolic acid decarboxylases (PADs).^9,10^

Previous research identified a thermally stable ancestor of phenolic acid decarboxylase (PAD N31, PDB: 8B30) derived from *Bacillus subtilis* (*Bs*PAD, PDB: 2P8G) through ancestral sequence reconstruction. Mytrollari et al. reported that PAD N31 exhibits an unfolding temperature of 78.1 °C, which is 20 °C higher than that of the wild-type *Bs*PAD, and an extended half-life time of 45 h at 60 °C, compared to less than 1 min for *Bs*PAD. These characteristics render PAD N31 a promising candidate for decarboxylations at elevated temperatures.^10^

Apart from finding thermostable biocatalysts, to transfer decarboxylations from the laboratory scale to an industrially relevant basis (>100 g/L), substrate solubility in the reaction media must be addressed (due to low solubilities of ∼0.92 g/L for ferulic acid or ∼1.23 g/L for caffeic acid in water, at room temperature).^11,12^ Biocatalysis in nonconventional media (BNCM) (e.g., solvent-free, organic solvent, supercritical fluids, ionic liquids, or deep eutectic solvents) offers alternatives in increasing the solubility of hydrophobic substrates, and our group has been particularly active in combining (biogenic) hydrophobic media with biorefinery-like approaches, establishing synthetic concepts at high substrate loadings and conversions (e.g., >100 g/L) in different nonconventional media.^13–15^

Among these nonconventional options for biocatalysis, DESs are formed by the interaction of hydrogen bond donors (HBDs) and hydrogen bond acceptors (HBAs).^16–19^ DESs offer ample design space to create solvents that simultaneously meet the requirements of the enzyme (i.e., activity and stability) and the process (i.e., substrate solubility, multi- and single-phase systems), while being biogenic at the same time. Customized DESs may be generated by varying the individual components and their molar ratios.^18,20^ DESs have shown applications in biocatalysis with oxidoreductases,^15,21–28^ transferases,^29,30^ hydrolases,^31–36^ photodecarboxylases,^37^ and lyases.^10,36,38,39^

Apart from selecting an appropriate reaction medium for process intensification, another major constraint for industrial scalability is the enzyme recovery and recycling. Enzyme immobilization has been implemented since the 1960s,^40^ as the heterogenization allows biocatalyst recyclability, enzyme-free product streams, and simplified downstream. Additionally, it enables the use of enzymes in continuous processing.^41–46^ The three main immobilization methods include entrapment, cross-linking, and covalent attachment.^47,48^ In particular, covalent immobilization benefits from strong interactions, yielding long shelf lives and preventing enzyme deactivation under extreme conditions (high temperature, nonaqueous solvents, and extreme pH).^49,50^ However, confining the enzyme within or onto a support matrix introduces new challenges, particularly mass transfer or diffusional limitations. Substrates must diffuse through the bulk solvent, into the film diffusion layer, and through the porous support to reach the active site. This inherent limitation, together with the need to handle a solid phase, makes the optimal reactor design key for success. A promising option for heterogeneous biocatalysis is the use of rotating bed reactors (RBRs), with many relevant examples.^13,46,51–55^

Notably, our research group has utilized RBRs for the scale-up of lignin-based substrate valorization in CPME to deliver 1 kg of 4-acetoxy-3-methoxy-styrene (4-AMS) with excellent yields (95%) in a 10 L RBR.^13^

In addition to the challenges associated with the scale-up of the upstream unit, the target product must be isolated from the complex reaction mixture, including removal of the catalyst, solvent, and unreacted substrate. Von Langermann and co-workers have applied different unit operations for downstream processing in biocatalysis,^56^ such as liquid–liquid extractions,^57–59^ adsorptive strategies,^60,61^ crystallization, or precipitation.^56,62^ Herein, an open challenge when using DESs is the subsequent downstream due to their low volatility, high viscosity, and the high solubility of various substrates. Strategies employed include antisolvents, crystallization, membrane filtration, solid–liquid extraction, liquid–liquid extraction, and short-path distillation.^63–65^ In any case, product extraction with organic solvents remains the most commonly reported strategy, often followed by solvent distillation, product recovery, and DES (and solvent) recycling.^66^ Other downstream strategies for DESs may include crystallization^67^ or solid–liquid extraction.^68^ Nevertheless, the reuse of DESs must be addressed and is still underrepresented in the literature, with few examples (e.g., Panić et al. showed the recovery of a DES (ChCl-Gly with 30 vol % water) after the bioconversion of 1-(3,4-dimethylphenyl)­ethenone (DMPA)).^69^

Following these thoughts, demonstrating that DESs could be used under industrial conditions for lignin-based derivatives would open new degrees of freedom and tunability options. Herein, Schweiger et al. used wild-type *Bs*PAD in 50 vol % ChCl-Gly (1:2) to reach 77% conversion of 400 mM caffeic acid (∼72 g/L) within 24 h (Scheme 1, Schweiger et al.).^38^ Concurrently, Myrtollari et al. demonstrated the advantages of the ancestor PAD N31 by further improving the conversion to 80% of 400 mM ferulic acid (∼77 g/L) in 70 vol % ChCl-Gly (1:2) at 60 °C (Scheme 1, Myrtollari et al.).^10^ We recently reported the effects of hydrophilic DESs, namely choline chloride–glycerol (ChCl-Gly, 1:2), choline chloride–ethylene glycol (ChCl-EG, 1:2), choline acetate–glycerol (ChAc-Gly, 1:2), and betaine–glycerol (Bet-Gly, 1:2) mixed with 20 vol % of phosphate buffer in terms of (i) substrate loading, (ii) enzyme activity, and (iii) enzyme stability. The process showed up to 14-fold higher ferulic acid solubility (43 mM, 9.7 g/L, in 80 vol % Bet-Gly) and 11 °C higher unfolding temperatures of PAD N31 (85.7 ± 3.0 °C) compared to pure buffer (75 °C, 50 mM KPi, pH 6). Performing the reaction at 1 M ferulic acid (200 g/L) resulted in 90% conversion using 80 vol % Bet-Gly (1:2) at the 1 mL scale (Scheme 1, Vaupel et al.).^12^

**Scheme 1:**
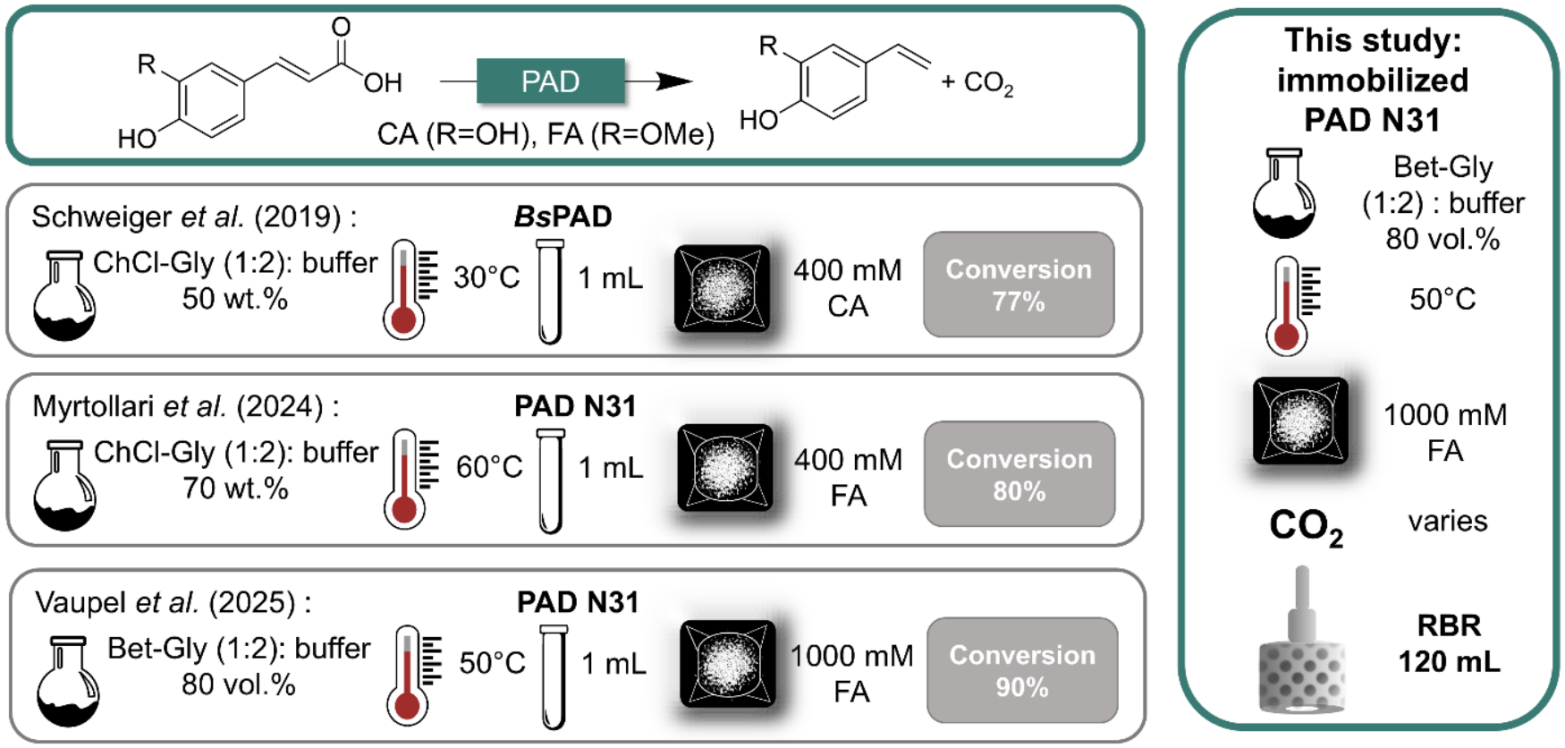
Reaction Scheme of the Decarboxylation of Ferulic Acid with Phenolic Acid Decarboxylase (PAD, Green Box at the Top)^a^

a Decarboxylation reactions carried out in DES/buffer mixtures to date (gray boxes) compared to the scale-up in this work (green box at the right-hand side). PAD: phenolic acid decarboxylase, CA: caffeic acid, FA: ferulic acid, ChCl: choline chloride, Gly: glycerol, Bet: betaine, and RBR: rotating bed reactor.

On this basis, the PAD-catalyzed decarboxylation of ferulic acid can be efficiently conducted with free (thermostable) enzymes at intensified conditions (molar scale). However, the transition from the mL scale to larger volumes is not trivial.^13^ When DESs are used and high substrate loadings are intended, the formation and retention of stoichiometric amounts of (biogenic) CO_2_ cannot be overlooked. Moreover, although applications of biocatalysis in DESs have been widely reported, the transition to a larger scale to demonstrate the following steps *en route* to industrial applications remains scarce. In RBRs, for instance, only one recent work studied enzymes and DESs for the reduction of halogenated aryl ketones (HAKs) in 30 vol % betaine–glucose–H_2_O (5:2:13) in PBS buffer (1 M, pH 6.0) with high yields (98%) and improved volumetric productivities (3.84 vs 1.20 g L^–1^ h^–1^) compared to a stirred tank reactor.^70^ Importantly, besides demonstrating the scalability in RBRs, assessing limitations and realistic options for the downstream in DES-based enzymatic reactions is also of utmost importance when scale-up systems are envisioned.

Taking all these considerations into account, this work investigates the scale-up for the decarboxylation of ferulic acid in RBRs, focusing on (i) PAD immobilization to ensure that enzymes can compete with tight economics of bulk commodities; (ii) evaluation of the prescalability using RBRs, assessing mass transfer limitations, and in particular the influence of CO_2_ in the DES phase; (iii) implementation of fed-batch strategies, to further optimize the system (Scheme 1, green box, right-hand side); and (iv) addressing the downstream of the DES through extractive methods to selectively achieve 4-vinylguaiacol (4-VG). Overall, we expect to cover often overlooked areas in DESs and biocatalysis to provide a clearer understanding of the industrial potential, as well as the remaining limitations when scaling up.

## Results and Discussion

Our previous work reported the molar-scale PAD-catalyzed decarboxylation of ferulic acid in 80 vol % Bet-Gly (1:2) with 20 vol % KPi (50 mM, pH 6), using cell-free extracts containing the enzyme at the 1 mL scale. Excellent conversions (90%) in 4 h were achieved,^12^ corresponding to the industrially relevant range of 100 g/L product. Taking those data as a basis, scale-up challenges in DESs were tackled using a RBR of 120 mL, to enable a thorough mixing to (expectedly) reduce mass transport limitations associated with the high viscosity of Bet-Gly (1:2) (1331 mPa·s).

### Covalent Immobilization of PAD N31

Covalent immobilization was preferred over affinity-based methods because of the potential DES interference. For PAD immobilization, Petermeier et al. analyzed 11 carriers, including seven with covalent bonding via epoxy or amino functionalization and varying spacer lengths.^11^ Among these, the 8415F Amino C6 (Seplife EMC7120S) carrier, cross-linked with glutaraldehyde, appeared promising. Thus, we selected this carrier to immobilize PAD N31 but assessed diformylfuran (DFF) as a cross-linking agent instead of glutaraldehyde. Glutaraldehyde is conventionally used for covalent cross-linking, yet it poses environmental toxicity. DFF, which can be biogenically derived, appears as a less toxic and more sustainable alternative.^71^ Crude cell-free extract (CFE) containing ∼50% PAD N31 was immobilized at a ratio of 1:4 resin/CFE (25 mg·mL^–1^). Both carriers exhibited comparable performance, achieving immobilization yields of 85.0 ± 0.5% (carrier with glutaraldehyde as cross-linker) and 90.3 ± 0.7% (carrier with DFF as cross-linker). The corresponding activities were 31 ± 5 U g^–1^ carrier for glutaraldehyde and 32 ± 2 U g^–1^ carrier for DFF, resulting in similar activity yields of 36.4 ± 6% and 38.5 ± 2% (see Table 1).

**Table 1:** Immobilization of PAD N31 on Seplife EMC7120S (Pore ϕ, 200–400 [Å]) Carriers with Two Different Cross-Linking Agents^a^

cross-linking agent	immobilization yield [%]	specific activity [U·g_carrier_^–1^]	activity yield [%]
glutaraldehyde	85.0 ± 0.5	31 ± 5	36.4 ± 6.0
DFF	90.3 ± 0.7	32 ± 2	38.5 ± 2.0

a In both cases, 0.4 mM cross-linking agent was used and incubated at room temperature for 18 h in KPi buffer (50 mM, pH 6). Activity measured in KPi buffer (50 mM, pH 6) with 10 mg of immobilized enzyme and 10 mM FA with 5 vol.% DMSO.

Moreover, no enzyme leaching from the carriers was observed (see the Supporting Information (SI), Figure S5). Given its comparable performance and improved environmental properties, DFF was selected as a cross-linker. Since our primary focus was to assess the potential scalability of DES-based enzymatic processes, further optimizations in the immobilizing procedure were not followed. Based on our understanding of both the enzyme’s catalytic activity and its stability in DES-based media, we investigated parameters that may affect the scale-up of the reaction.

### Condition Screening

#### Closed and Open Systems

As stated above, the CO_2_ generated during the reaction may be relevant, as its accumulation is expected when DESs are used as media. Furthermore, one should consider not only the *innocent* accumulation of CO_2_ in DESs but also how CO_2_–DES interactions may give rise to changes in physical–chemical properties (analogous to an “expanded phase”),^72^ which may superimpose on the thermodynamic equilibrium of the reaction. Several studies have assessed the carbon-capture capacity of DESs. Lagoarušić et al. reported the solubility of CO_2_ in glycerol-based DESs with different buffer (50 mM KPi, pH 7.4) contents, finding that Bet-Gly achieved a CO_2_ solubility of 586 mg·L^–1^ with 30 wt % buffer.^73^ Ma et al. correlated the viscosity of DESs and their CO_2_ solubility, noting that higher viscosity impairs mass transfer and thus diminishes the solvent’s CO_2_ capture capability.^74^ Therefore, buffers may influence the solubility of CO_2_, as a balance must be struck between CO_2_ affinity and mass transport. Based on these insights, we investigated the potential effect of dissolved CO_2_ on the actual availability of ferulic acid in the system for enzymatic decarboxylation (Figure 1). It can be assumed that ferulic acid interacts with Bet-Gly via hydrogen bonds due to its carboxyl group, and therefore, its availability may also be influenced by CO_2_ accumulation within the DES network. To investigate this, we conducted reactions in both open and closed 10 mL reaction vessels at 50 °C with 300 mM (58 g/L) ferulic acid (Figure 1A). The final volume used in these reactions is 10-fold greater than the 1 mL starting volume used in our previous research.^12^ Likewise, total enzyme activity was reduced by 92% (to 14 U) compared to the 1 mL experiments, to ensure better comparability with the future reactions in the RBR, in which the (bio)­catalyst loading is limited by the basket size and the available amount of the immobilized enzyme. Therefore, we defined the ratio of the immobilized enzyme’s activity (U) to the amount of ferulic acid (mol) as the reference value used in all experiments (Table 2).

**Figure 1:**
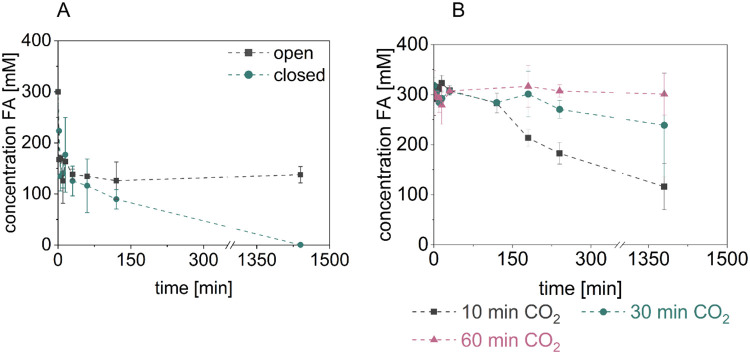
(A) Decarboxylation of 300 mM ferulic acid (FA) in 80 vol.% Bet-Gly (1:2) and 20 vol.% KPi (50 mM, pH 6) with 14 U of immobilized enzyme at 50 °C and 540 rpm in a closed and open system. (B) Different concentrations of CO_2_ inside the decarboxylation reaction of 300 mM ferulic acid with 14 U of immobilized enzyme at 50 °C and 540 rpm. The reaction volume was purged with pure CO_2_ at a volumetric flow rate of 219 Ncc/m for 10, 30, and 60 min at the beginning of the reaction. Experiments were performed in duplicates. Reliable quantification of 4-VG by HPLC was not possible due to interference from the FA–DES reaction system, which impaired clear, reproducible detection of the 4-VG signal. The reaction progress was therefore evaluated primarily based on the decrease in ferulic acid concentration.

**Table 2:** Summary of Reaction Conditions and Results Obtained in Screening Experiments Performed in 10 mL Glass Vials and in the 120 mL Rotating Bed Reactor (RBR) Using 80 vol.% Bet:Gly (1:2) and KPi Buffer^a^

experiment →	1.0	2.1	2.2	3.1	3.2	4.1	4.2	4.3	5.0
condition	free CFE	open	closed	open	closed	open			open
reaction vessel	glass-vial					rotating bed reactor (RBR)			
volume (mL)	1	10	10	10	10	120	120	120	120
buffer capacity (mM)	50	50	50	**1000**	**1000**	50	50	50	50
rotating speed (rpm)	640^b^	540^b^	540^b^	540^b^	540^b^	**500**	**600**	**800**	800
[FA] (mM) start	1000	300	300	300	300	1000	1000	1000	1000
CFE mass (mg)	0.9	---	---	---	---	---	---	---	---
activity (U)	698	14.1	14.1	14.1	14.1	573	573	601	287 + 287 + 228 sequential addition
ratio PAD N31 activity/[FA] (U/mol)	698000	4700	4700	4700	4700	4775	4775	5006	6672
time until max. conversion (h)	4	24	24	24	24	24	8	24	24
[FA] (mM) end	124	138	0.3	150	57	422	341	413	462
conversion (%)	**88**	**54**	**>99**	**50**	**81**	**58**	**66**	**59**	**53**

a Abbreviations: CFE, cell-free extract; RBR, rotating bed reactor; FA, ferulic acid; PAD N31, phenolic acid decarboxylase N31; Bet, betaine; and Gly, glycerol. “Open” and “closed” denote experiments conducted with or without gas exchange, respectively. Experiment 1.0 was carried out with free CFE; experiments 2.1–5.0 were performed with immobilized PAD N31. The table reports the reactor type, reaction volume, buffer capacity, rotation speed, nominal initial FA concentration ([FA]_start_), enzyme loading, activity-to-substrate ratio, time required to reach maximum conversion, measured final FA concentration ([FA]_end_), and final conversion. Conversion was determined from the decrease in FA concentration. In experiment 5.0, enzyme beads were introduced sequentially during basket exchange (287 U at 0 min, 287 U at 120 min, and 228 U at 240 min; total enzyme loading: 801 U). Experiments 1.0, 2.1–2.2, and 3.1–3.2 were conducted in a thermoshaker at the indicated rotational speed.

b The reaction was carried out in a thermoshaker at the respective rpm.

Reactions with an open lid achieved ∼46% conversion within 2 h (Figure 1A, Table 2, 3.1), whereas the closed-lid reaction reached complete conversion within 19 h (Figure 1A, Table 2, 2.2). An experiment using an additional buffer did not yield higher conversions (see the SI, Figure S6), ruling out the possibility that the reaction in the open vessel was impaired by water loss. Thus, different CO_2_ concentrations inside the reaction system were assessed by gassing vials with pure CO_2_ at a volume flow of 219 Ncc/m over three different periods of time (10–60 min) at the start of the reaction. Figure 1B shows that the reaction proceeds more effectively when CO_2_ is gassed for 10 min (60% conversion), thereby increasing conversion slightly relative to the open system (Figure 1A). Notably, the reaction exhibited lower conversion rates at longer CO_2_ gassing times. Thus, when CO_2_ was gassed for 30 min, a 25% conversion was achieved after 24 h, whereas CO_2_ application for 60 min resulted in almost no conversion (∼1%).

A certain amount of CO_2_ captured/accumulated in the system may increase the FA availability within the DES network for the enzyme. Still, the absence of enzyme activity during continuous CO_2_ addition suggests that a critical CO_2_ concentration must not be exceeded (Figure 1B). Overall, the results suggest an optimal CO_2_ concentration that maintains an adequate FA availability without quenching the reaction. This optimal CO_2_ level can be achieved in a closed system (Figure 1A) and in an open system via brief CO_2_ gassing at the start of the reaction (Figure 1B). Therefore, process development with scale-up in mind indicates that DES media are more complex than conventional solvents and careful optimization is required at each step.

#### Buffer Capacity: pH Influence

The dissolution of CO_2_ in water produces carbonic acid and can lead to acidification and subsequent enzyme denaturation. Using buffers generally stabilizes the system and reduces pH fluctuations resulting from changes in FA solvation or reaction progress (from carboxylic acid to decarboxylated product). Pesci et al. (using *Mycobacterium colombiense* PAD) demonstrated a positive correlation between buffer capacity and conversion rates in a two-phase system of an enzyme in buffer and FA in hexane, as higher buffer capacity increases FA solubility, thereby facilitating its transfer into the enzyme solution.^75^ When transitioning to a single-phase DES buffer system (ChCl-Gly, 70%, buffer 30%), earlier studies by Myrtollari et al. (2024, PAD N31) also showed a positive correlation between buffer capacity and conversions.^10^ Petermeier et al. employed 500 mM buffer at pH 7.3 to facilitate higher FA solubility.^11^ However, the reaction ceased as FA consumption raised the pH beyond the enzyme’s optimal range. Additionally, the authors examined the effect of water activity on the reaction, finding that controlled addition of water to the immobilized PAD and CPME system significantly increased FA conversion.^9,13^ For the single-phase DES system used in this study, −80 vol % DES (Bet-Gly (1:2)) and 20 vol % buffer solution −, the goal was to assess whether an increase in buffer capacity would lead to higher conversions. Therefore, both the open and closed systems were compared with buffer concentrations of 50 mM and 1 M (Figure 2).

**Figure 2:**
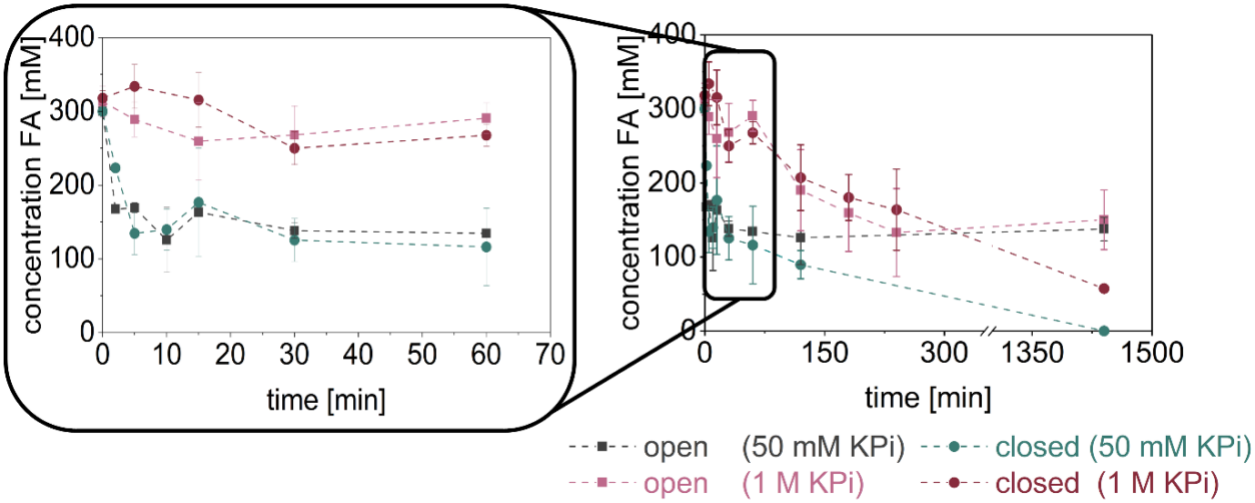
PAD-catalyzed decarboxylation of 300 mM (58 g/L) FA in 80 vol % Bet-Gly and 20 vol % KPi (50 mM or 1 M, pH 6) with 14 U of immobilized enzyme at 50 °C and 540 rpm in a closed and open system.

Although the initial stages of both reactions show that conversions of low-concentrated buffers are slightly higher (Figure 2), this is attenuated after 24 h for the open reactor configuration (Figure 2). However, the closed reactor with 1 M buffer did not reach full conversion, whereas the closed reactor with 50 mM buffer achieved almost full conversion within 24 h (Figure 2). These findings contrast with the above-mentioned studies, in which higher buffer concentrations improved overall conversion in an open reactor system. An explanation here may be that the higher buffer capacity dissolves more CO_2_, and as a result, CO_2_ is no longer available in sufficient concentrations within the DES components, reducing FA availability (due to the interaction of CO_2_ with the DES network vs the interactions with FA; see discussion above). Thus, a buffer capacity of 50 mM was chosen for all subsequent investigations, and a pH measurement confirmed that the reaction proceeded without a significant change in the pH value (see the SI, Figure S7).

### Transferring the Reaction System to the RBR

The reaction was subsequently transferred to a 120 mL RBR (a 12-fold increase compared with 10 mL reactions). RBRs offer superior mixing capabilities compared to other stirred-tank reactors and are particularly effective for viscous solutions.^76,77^ Immobilized PAD N31 was used in the RBR employing slurry reaction conditions with high ferulic acid loading (1 M, 200 g/L).

#### Stirring Rate

Using immobilized biocatalysts, mass transfer limitations primarily arise from pore (internal) diffusion within the carrier and from film (external) diffusion to the carrier. Although the carrier particle size, which influences pore diffusion and total surface area, was fixed by the manufacturer’s specifications, we aimed to minimize film diffusion. The high viscosity of the DES (Bet-Gly (1:2)) significantly intensifies external mass transport limitations, thereby promoting the formation of a laminar boundary layer between the carrier and the bulk solution. To tackle this, the stirring speed of the RBR was increased to reduce the thickness of the stagnant film layer and enhance the substrate transport rate to the immobilized enzyme surface. The reaction was carried out at stirring speeds of 500, 600, and 800 rpm (Figure 3).

**Figure 3:**
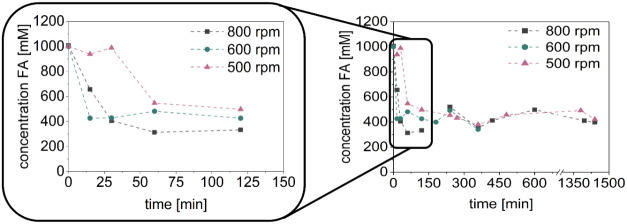
Progressive curve of the decarboxylation of 1 M (194 g/L) ferulic acid in an RBR with different stirring speeds. Reaction conditions: 120 mL reaction volume containing 1 M FA and 573 U of immobilized PAD N31 at 50 °C and different stirring speeds (500, 600, and 800 rpm). The reaction conditions are reported in Table 2. Lines connecting experimental data are for illustrative purposes only.

Increasing the stirring rate led to faster reactions (Figure 3), and at 800 and 600 rpm, the conversion plateaued within 1 h, whereas longer times were required at 500 rpm. Notably, all reactions led to constant conversions of ∼58–66% (Figure 3) in a similar trend (Table 2, 4.1–4.3). The reactor is an open system, comparing the RBR reaction with the open vial reaction showed that RBRs performed 1.5-fold better with 46% conversion in the vial and 66% in the RBR (600 rpm) (Table 2, 2.1, 4.2). This indicates that the reaction can be effectively scaled up, although full conversion is not achieved, as observed in our previous work at the 1 mL scale.^12^

The impact of CO_2_ dissolution in the DES phase creates complexities compared to other *classic* solvents such as CPME. Scale-up reactions in DESs, expectedly an upcoming research avenue in synthetic chemistry, must be addressed holistically to consider all possible cross-reactivities between DES components, water, reagents, and (by)­products such as (accumulated) CO_2_. Subsequently, an experiment using CO_2_ gas was conducted in the RBR. A sparger was integrated into the reactor to facilitate continuous CO_2_ addition. However, in this setup, the reaction was inhibited by excessive CO_2_, resulting in no conversion within a 24 h period (see the SI, Figure S8). This confirms the results obtained in the 10 mL-scale reaction, where gassing might increase the concentration of available nominal ferulic acid, but at the same time, the overabundance of CO_2_ would prevent any conversion. In addition, a fed-batch approach was carried out at the 120 mL scale with 890 mM ferulic acid and 573 U of immobilized PAD N31 at 50 °C and 600 rpm. The substrate was added in three different batches (0 min: 333 mM, 60 min: 333 mM, and 120 min: 249 mM), but no further increase in conversion was observed (see the SI, Figure S9). Since the reaction mixture already formed a slurry, the system had effectively reached the solubility limit of ferulic acid under the applied conditions.

#### Sequential Enzyme Addition in the RBR

Subsequently, the total enzyme loading was increased by 28%, from 573 U to 801 U, and the enzyme was aggregated via a sequential addition protocol. Enzyme beads were added at 0, 120, and 240 min (indicated by arrows in Figure 4A). FA concentration increased in each basket exchange/enzyme addition (Figure 4A, Table 2, 5.0), and after completion of the mixing process for basket replacement, the measured concentration dropped again. This effect is most likely attributable not to the enzyme addition itself, but to the brief mechanical disturbance and altered mixing conditions during basket replacement. Since the reaction medium already formed a slurry, indicating operation close to the solubility limit of FA, this handling step may have temporarily changed the measurable FA concentration by redistributing suspended or locally concentrated substrate fractions. After completion of the basket exchange and renewed homogenization, the measured FA concentration decreased again. In any case, despite the increased overall enzyme loading, conversions remained unchanged at ∼60%, further supporting the hypothesis that FA may become more or less available in the DES system depending on the conditions. A comparison with the control experiment, initiated with the full 573 U loaded at *t* = 0 min (Figure 4B, Table 2, 4.3), demonstrated a comparable kinetic progression and a slightly higher final conversion of 59%. As expected, the control reaction (initial loading: 573 U) reached the final conversion faster (∼120 min) than the sequential addition protocol (initial loading: 286.5 U, reaching ∼240 min).

**Figure 4:**
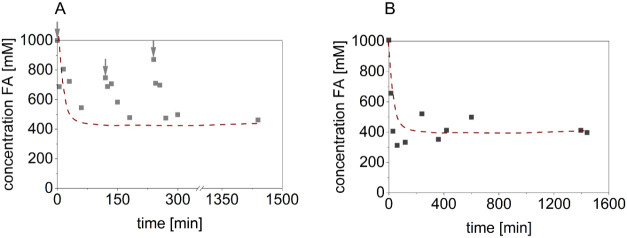
Fed-batch enzyme addition to the reaction in the RBR. Reaction conditions: 120 mL reaction volume containing 1000 mM FA and immobilized PAD N31 at 50 °C and 800 rpm. (A) Basket was changed three times at time points 0, 120, and 240 min, yielding a total amount of PAD N31 of 801 U (indicated by the arrows). (B) Basket was only added at the beginning of the reaction with a total amount of PAD N31 of 573 U. Lines connecting experimental data are for illustration purposes.

Summarizing all the findings, the reaction in a 1 mL vial reached 88% conversion at 1 M FA (Table 2, 1.0), and when scaled up to 10 mL (Table 2, 2.1–3.2), the highest conversion for 300 mM was >99% in a closed reaction vessel with 50 mM buffer (Table 2, 2.2). Further scaling to the RBR (120 mL) resulted in conversions between 53 and 66% (Table 2, 4–5), with the best performance achieved at 600 rpm in an open reactor system (Table 2, 4.2). The maximum conversion of 66% at a stirring speed of 600 rpm was achieved in RBR reactions within 4 h, whereas at 800 rpm, the reaction terminated after 1 h at 59%, indicating faster kinetics and overcoming mass transport limitations. Overall, the results demonstrate the complexity of scaling up reactions in DES-aqueous media and the need to carefully understand each (sub)­reaction occurring, from substrate dissolution/availability to CO_2_ accumulation, acidification, and enzyme stability. The potential of DESs as tunable media must also be studied at larger scales and in other synthetic systems to generate knowledge that can benefit from the potential of these solvents.

### Optimization of the Downstream Processing from the DES

While DESs result in useful media for (bio)­catalysis (upstream), as shown at the laboratory scale, research on how to implement solid downstream alternatives has been scarce hitherto. Selective liquid–liquid separations in which DES components are not extracted with the product would be optimal. To address this, the miscibility of Bet-Gly (1:2) in five organic solvents, 2-MeTHF, methyl *tert*-butyl ether (MTBE), CPME, EtOAc, and heptane, was assessed, and all of them resulted in immiscibility (see the SI, Figures S10–S11). Subsequently, the partition coefficients (log­(*K*)) for FA and 4-VG for a biphasic system composed of a DES (80 vol % Bet-Gly (1:2) with 20 vol % KPi) and the organic solvents were measured. With respect to ferulic acid, 2-MeTHF exerted the highest solubility (165 g/L), while CPME, MTBE, and EtOAc displayed comparable performance at a lower level (16 and 17 g/L (30 °C) and 26 g/L (25 °C)).^11,12,78^ Heptane did not dissolve ferulic acid, indicating a strong correlation between extraction efficiency and solvent polarity.^11^ For biphasic DES–solvent media, the partition coefficient (log *K*) of ferulic acid was determined by HPLC, with 2-MeTHF exhibiting the highest extraction efficiency (see the SI, Figure S12), which correlates with the observed FA solubility. A precise partition coefficient could not be determined for 4-VG due to its immiscibility with the aqueous mobile phase used in the HPLC, and thus, qualitative analysis was conducted. Again, 2-MeTHF showed the highest extraction capacity for 4-VG but also efficiently extracted FA, indicating a need for an additional separation step to isolate 4-VG selectively if 2-MeTHF is used as a liquid–liquid extraction solvent, and conversions are not complete.

Overall, this requires more solvent volume during the downstream processing. MTBE, CPME, and EtOAc yielded similar extraction efficiencies for 4-VG but were not selective for ferulic acid (Table 3). Conversely, heptane, the nonpolar solvent, selectively extracted 4-VG. The experimentally determined log *K* values show the same trend as the partition coefficients calculated by Petermeier et al. of the five organic solvents and water at 30 °C. The calculated log *K* showed for 2-MeTHF a partition coefficient of approximately 2.9 for ferulic acid and 3.7 for 4-VG. For MTBE and CPME, similar log *K* values were calculated, approximately 2.1 and 1.9 for ferulic acid and 3.6 for 4-VG. No log *K* values could be calculated for EtOAc, whereas *n*-heptane displayed a partition coefficient of ferulic acid of −1.1 and that 4-VG of 2.5.

**Table 3:** Examined Miscibility of Bet-Gly (1:2) and log *K* of Different Organic Extraction Solvents for Ferulic Acid and 4-Vinylguaiacol^a^

solvent	miscibility (Bet-Gly (1:2)) at 20 °C	log *K* (ferulic acid)	qualitative log *K* ^b^ (4-vinylguaiacol)
2-Me-THF	immiscible	0.2	+++
MTBE	immiscible	–0.5	++
CPME	immiscible	–0.3	++
heptane	immiscible	not possible	+
EtOAc	immiscible	–0.2	++

a 2-MeTHF = 2 methyltetrahydrofuran, MTBE = *tert*-methyl-butyl ether, CPME = cyclopentyl methyl ether, EtOAc = ethyl acetate, Bet = betaine, and Gly = glycerol.

b Qualitative measurements were carried out by comparing integration areas in the HPLC measurements. Exact quantification was not possible because of the extreme hydrophobicity of 4-VG.

While the solvents analyzed were superior at extracting 4-VG, they would require further downstream processing to separate residual FA from 4-VG (unless full conversion was achieved). In contrast, heptane provided optimal selectivity, and a direct extraction was implemented following the sequential enzyme addition in the RBR system. The DES buffer system was extracted with a 2:1-ratio heptane:DES buffer system, dried over MgSO_4_, and then evaporated under reduced pressure. 4.4 g of product (corresponding to a 29% yield, nonoptimized conditions) was isolated at high purity (>99% HPLC [with heptane traces in NMR], see the SI, Figures S13 and S14), thereby eliminating subsequent purification requirements, which would imply less solvent used in the overall process. The approach may be optimized in terms of yield, and heptane may be considered a “usable” solvent in synthetic chemistry.^79^ Nevertheless, the quest for other biogenic alternatives that may be more neutral in carbon emissions remains in our group, to identify conditions in which downstream and sustainability can be better met.

## Conclusions

Most of the examples of biocatalysis in DESs have been conducted at a small scale, for proof-of-concept purposes. The importance of assessing the technical feasibility and potential challenges associated with the scale-up of DESs’ biocatalytic processes has been somewhat overlooked. The same applies to the downstream unit, with only a few examples disclosed. Taking these premises into account, this paper has explored the potential of scaling up the decarboxylation of ferulic acid in 80 vol % Bet-Gly (1:2) in an RBR for the valorization of lignin-derived materials. After enzyme immobilization using diformylfuran (DFF) as a cross-linker, processes achieving full conversion at 300 mM ferulic acid (∼58 g/L) on the 10 mL scale were demonstrated. However, challenges arose when RBRs at 120 mL were established with higher substrate loadings (up to 1 M, ∼200 g/L). In those RBRs, increasing the stirring speed shortened the reaction time (e.g., from 1 h at 800 rpm), but the conversion plateaued at ∼60%. One factor associated with this may be the accumulation of CO_2_ in the DES media, which would not happen in other classic solvents, which may improve/hinder the availability of ferulic acid in the reaction. For the downstream processing, heptane provided optimal selectivity. Leveraging this selective solvent, a direct extraction implemented immediately following the sequential enzyme addition in the RBR system successfully isolated 4.4 g of product (corresponding to 29% yield) at high purity (>99% HPLC [with heptane traces in NMR]). Although heptane is regarded as an “acceptable” solvent in many rankings, identifying other, more benign solvents (e.g., biogenic options) remains a task for future research in our group.

Beyond the results reported in this work, a more general observation is the need for further research on DESs and biocatalysis at larger scales. The potential of tunable solvents has now been widely recognized, as they can be enzyme-compatible and excellent substrate solubilizers simultaneously. However, DESs do not behave as classic solvents, and unexpected challenges can appear in the scale-up, especially when high substrate loadings, leading to high product titers, are considered. The same may apply to downstream activities, which are now emerging as a new frontier in research on DES and enzymatic reactions. Generating knowledge in these areas will be crucial to validate whether DESs can find their place in future industrial (sustainable) applications by untapping their tremendous potential.

## Supplementary Material



## Notes

Reactor cultivation, SDS PAGE analysis, HPLC chromatograms, calibration curve, 10 mL vessel reaction with 20% more buffer, pH measurement, RBR reaction with continuous CO_2_ addition, sequential enzyme addition in the RBR, NMR spectra (PDF)

## Abbreviations

4-VG: 4-vinylguaiacol
Bet: betaine
DES: deep eutectic solvent
FA: ferulic acid
Gly: glycerol
PAD: phenolic acid decarboxylase
RBR: rotating bed reactor
