# Supplementary material for: Scaling Up Is Not the Same but Bigger: Overcoming (Some) Limitations in Enzymatic Decarboxylations in Rotating Bed Reactors in Deep Eutectic Solvents
Source: Org Process Res Dev. 2026 May 28;30(6):1652–61. doi: 10.1021/acs.oprd.6c00074 (PMC13289501; doi:10.1021/acs.oprd.6c00074)
Supplement: Supplementary file 1 [file op6c00074_si_001.pdf]

Supporting Information

Scaling-up is not the-same-but-bigger: Overcoming (some) limitations in enzymatic decarboxylations in rotating bed reactors in Deep Eutectic Solvents

Sonja Vaupel[a], Lars-Erik Meyer[a], Pablo Domínguez de María[b] and Selin Kara*[a], [c]

[a] S. Vaupel; L.-E. Meyer; S. Kara*

Institute of Technical Chemistry, Leibniz University Hannover, Callinstr. 5, 30167 Hannover, Germany

*E-mail: selin.kara@iftc.uni-hannover.de

[b] P. Domínguez de María

Sustainable Momentum, SL. Av. Ansite 3, 4-6, 35011, Las Palmas de Gran Canaria, Canary Islands, Spain

[c] S. Kara

Biocatalysis and Bioprocessing Group, Department of Biological and Chemical Engineering, Aarhus University, Gustav Wieds Vej 10, 8000 Aarhus C, Denmark

E-mail: selin.kara@bce.au.dk

Table of contents

1. MATERIALS & METHODS 3

1.1. Expression of PAD N31 in fed-batch bioreactor cultivation 4

1.2. High-performance liquid chromatography 6

1.3. Covalent immobilization of PAD N31 7

1.4. Activity assay of free CFE in aqueous media 7

1.5. Protein assay 7

1.6. Activity assay of immobilized CFE in aqueous media 8

1.7. Reaction intensification in the rotating bed reactor 8

1.8. Miscibility studies 8

1.9. Extraction studies and determination of log K values 8

1.11. CO_2 supplementation protocol 9

1.12. Smaller-scale reaction with higher buffer capacity or higher buffer content 9

2. ADDITIONAL INFORMATION 10

2.1. Reactor cultivation of PAD N31 10

2.2. SDS-PAGE analysis of the CFE of PAD N31 11

2.3. Exemplary HPLC chromatograms and calibration of ferulic acid in different DESs 12

2.4. Leaching experiments of PAD N31 from the carrier in DES 12

2.5. 10 mL vessel reaction with 20% more buffer 13

2.6. pH measurement after reaction 13

2.7. RBR reaction with continuous CO_2 addition 14

2.8. Sequential enzyme addition in the RBR 15

2.9. NMR spectra of Bet-Gly 16

2.10. NMR spectra of extraction solvents after contact with DES 18

3. REFERENCES 27

1. Materials & Methods

All chemicals, materials and solvents were obtained from commercial suppliers (Acros Organics, Alfa Aesar, Sigma-Aldrich, VWR International, Carl-Roth GmbH, Merck KGaA, Thermo Fisher Scientific, TCI Europe, Biowest, abcr GmbH, Biozym Scientific GmbH) and used as received: *trans*-ferulic acid (FA, ≥99% grade), K_2HPO_4 (≥99%), KH_2PO_4 (≥99%), NaOH (≥98%, p.a.), aqueous HCl (37%), dimethyl sulfoxide (DMSO, ≥99.5%), choline chloride (ChCl, 99%), glycerol (Gly, 99%), ethylene glycol (EG, ≥98.0%), betaine (Bet, ≥99.0%), choline acetate (ChAc, 98%), Karl-Fischer solvent CM (Art. No. 85461.290), bovine serum albumin (BSA, 2 mg/mL standard), formic acid (≥95%), $MgSO_4 \cdot 7\ H_2O$ (≥99.0%), D(+)-glucose monohydrat (≥97.5%), $Na_2MoO_4 \cdot H_2O$ (≥99.5%), $CoCl_2 \cdot 6\ H_2O$ (≥96.0%), $MnCl_2 \cdot 4\ H_2O$ (≥99.0%), $CuCl_2 \cdot 2\ H_2O$ (≥99.0%), H_3BO_3, $Zn(CH_3COO)_2 \cdot 2\ H_2O$ (≥98.0%), ethylenediamine tetraacetic acid disodium salt dihydrate (Titriplex III, ≥99.0%), $(NH_4)_2HPO_4$ (≥98.0%), citric acid, Fe(III) citrate. Acetonitrile (≥99.95%, UHPLC grade) used for HPLC was purchased from VWR International.

1.1. Expression of PAD N31 in fed-batch bioreactor cultivation

The reactor cultivation was carried out in a simple, defined autoinduction medium using T7-based *Escherichia coli* strains after Li *et al.* (2011).[1]

Table S1. Composition of stock solutions for the reactor cultivation.

Name	Working concentration	Sterilization method
50x $MgSO_4$ stock solution	0.586 g·L^{-1} $MgSO_4$	121 °C autoclave
20x glucose stock solution	10.91 g·L^{-1} glucose	121 °C autoclave
5000x $Na_2MoO_4·H_2O$ stock solution	2.1 mg·L^{-1} $Na_2MoO_4·2\ H_2O$	sterile filtration (0.2 μm)
2000x trace element stock solution	2.5 mg·L^{-1} $CoCl_2·6\ H_2O$<br>15 mg·L^{-1} $MnCl_2·4\ H_2O$<br>1.5 mg·L^{-1} $CuCl_2·2\ H_2O$<br>3 mg·L^{-1} H_3BO_3<br>33.8 mg·L^{-1} $Zn(CH_3COO)_2·2\ H_2O$<br>14.10 mg·L^{-1} Titriplex III	sterile filtration (0.2 μm)
50x $(NH_4)_2HPO_4$	4 g·L^{-1} $(NH_4)_2HPO_4$	121 °C autoclave or sterile filtration (0.2 μm)
10x KH_2PO_4	13.3 g·L^{-1} KH_2PO_4	121 °C autoclave or sterile filtration (0.2 μm)
50x citric acid stock solution	1.5542 g·L^{-1} citric acid	121 °C autoclave or sterile filtration (0.2 μm)
50x Fe(III) citrate stock solution	0.1008 g·L^{-1} Fe(III) citrate	121 °C autoclave)
5 mol L^{-1} NaOH stock solution	To bring final pH to 6.8	

Table S2. Composition of stock solutions for the Defined Non-inducing Broth (DNB).

Groups	Name of medium		Defined Non-inducing Broth (DNB)
	Volume of stock solution		mL
A group	Magnesium*	50x $MgSO_4$ stock solution	20
	Carbon source*	20x glucose stock solution	50
	Trace**	5000x $Na_2MoO_4 \cdot H_2O$ stock solution	0.2
B group	Elements**	2000x trace element stock solution	0.5
	Nitrogen*	50x $(NH_4)_2HPO_4$	20
	Phosphate and other salts*	10x KH_2PO_4	100
		50x citric acid stock solution	20
		50x Fe(III) citrate stock solution	20
	pH adjusting*	50x 5 mol L^{-1} NaOH stock solution	13.5
	Solvent*	Water (Mili-Q)	756.5
**A group:** Mix magnesium and carbon source solution, sterilize. After adding 5000x $Na_2MoO_4 \cdot H_2O$ stock solution, the solution (A group) can be kept for long term storage at room temperature. **B group:** Mix nitrogen, phosphate and other salts and add solvent, adjust to pH 6.8, sterilize. After adding 2000x trace element stock solution (B group) can be kept for long term storage at room temperature. * autoclave (or sterilize by filtration); ** sterilize by filtration.			

The precultures were carried out in 20 mL DNB-Medium supplemented with 50 $\mu g \cdot mL^{-1}$ Kanamycin in a 100 mL baffled shake flask. Precultures were inoculated from glycerol stocks of *E. coli* BL21 (DE3) carrying the pET-28a(+) plasmid (with the PAD N31 gene) and incubated overnight at 37 °C and 150 rpm on an orbital shaker. The main cultures were conducted in a 2 L stirred-tank bioreactor (Biostat A+, Sartorius) with a working volume of 1.5 L of DNB

medium supplemented with 50 µg·mL^{-1} kanamycin. The bioreactor was equipped with two Rushton impellers. The reactor was gassed with compressed air with an aeration rate of 1 vvm. The stirrer speed is cascaded (200–1000 rpm) so that the oxygen partial pressure (pO2) in the culture does not decrease below 30%. Foam production was controlled by the addition of antifoam. The pH was set to 7 and maintained by titration with 1 M HCl and 25% ammonia. The bioreactor was inoculated with preculture to give an initial OD_{600} of 0.1 rel. AU. Fermentation was carried out at 37 °C until induction.

Glucose feed was initiated after the end of the batch phase, as indicated by a peak in the DO signal as the initially added carbon source was depleted. The feed contained 129.89 g/200 mL (65%) glucose in water, 100 µL trace element solution, 40 µL Na_2MoO_4 solution, 1.183 mL Fe(III) citrate solution, 4 mL $MgSO_4$, and 50 µg·mL^{-1} kanamycin with a feed rate starting at 180 µL/min with an approximate logarithmic increase until 416 µL/min.

Protein overproduction was induced after 18 h by the addition of 0.5 mM isopropyl-ß-thiogalactopyranoside (IPTG). The gene overexpression was carried out for 5.5 h. Subsequently, cells were harvested by centrifugation (5,000 rpm for 30 min at 4°C) and suspended in KPi buffer (50 mM, pH 6). The cells were disrupted by high-pressure homogenization (15,000 psi, 3 cycles), and the cell debris was removed by centrifugation (5,000 rpm, 50 min, and 4°C). The supernatant was filtered through a bottle-top filter with a 0.22 µm pore size.

1.2. High-performance liquid chromatography

Both qualitative and quantitative analyses were performed using a Hitachi Chromaster high-performance liquid chromatography system (Japan) comprising a 5160 quaternary pump, a 5260 standard autosampler, a 5310 column oven, and a 5430 DAD detector. A Kinetex® 2.6 µm F5 Core-Shell LC column (50 × 4.6 mm, Phenomenex) was used. The sample injection volume was 10 µL, the column oven temperature 30 ± 0.5 °C, and the total flow rate 0.8 mL min^{-1}. A gradient HPLC method of 0.1% formic acid in water (A) and acetonitrile (B) was used. The initial mobile-phase composition was 95% A and 5% B, then changed to 20% A and 80% B over 5 min, and then held constant for 2 min. Afterward, the gradient declined within 1 min to the initial mobile-phase composition of 95% A and 5% B and remained stable for a further 7 min. The 310 nm channel of the diode array detector (DAD) was used for calibration and quantification of ferulic acid.

1.3. Covalent immobilization of PAD N31

The protocol was adapted from Petermeier *et al.*[2] Prior to enzyme immobilization, carrier beads (Amino C6 8415F, Seplife EMC7120S) were washed three times with KPi buffer (50 mM, pH 6.0) using a resin-to-buffer ratio of 1/1 (w/v). Next, the beads were incubated with glutaraldehyde / DFF buffer ($4.23x10^{-4}$ mol in KPi buffer, 50 mM, pH 6.0) in a resin-to-buffer ratio of 1/4 (w/v). The slurry was mixed for 1 h at room temperature using a self-made end-over-end mixer, after which the supernatant was removed, and the beads were washed 4 times with KPi buffer (50 mM, pH 6.0) at a resin-to-buffer ratio of 1/1 (w/v). A solution of 25 $mg·mL^{-1}$ crude CFE (protein content ~50%) was prepared in immobilization buffer (50 mM KPi, pH 6.0). Washed carrier beads were mixed with the crude enzyme solution at a resin-to-buffer ratio of 1/4 (w/v). The slurry was gently mixed for 18 h at room temperature on a self-made end-over-end mixer. Lastly, the mixtures were centrifuged (13,200 rpm, 1 min), and the supernatant was removed. The beads were washed twice with KPi buffer (50 mM, pH 6.0) at a resin/buffer ratio of 1/2 (w/v) each, and all supernatants (original and after both washing steps) were combined. The protein content of the collected liquid phase was determined using the reported protein assay. Wet immobilized enzyme preparations were stored in closed vessels at +4 °C.

1.4. Activity assay of free CFE in aqueous media

This standard assay was performed in KPi buffer (50 mM, pH 6.0) with ferulic acid as the substrate at an initial concentration of 10 mM, thereby ensuring zero-order kinetics. For the former, 900 µL KPi buffer was mixed with 50 µL FA stock (200 mM in DMSO) and heated to 30°C. To initiate the reaction, 50 µL of 1 mg/mL CFE was added, and the mixture was subjected to 30°C and 1200 rpm. Samples of each 40 µL were quenched and diluted by addition to 960 µL water/acetonitrile (1/1, v/v) and subjected to HPLC analysis. Standard sampling times were 0, 2, 3, 4, and 5 min. All derived kinetic data are based on substrate depletion.

1.5. Protein assay

To determine protein concentration, the Pierce™ BCA Protein Assay Kit was used according to the manufacturer's instructions. For the assay, 25 µL aliquots of the BSA standard or unknown sample (20–2000 µg/mL) were loaded into microplate wells in triplicate. Each well received 200 µL of the Working Reagent (WR), followed by 30 seconds of mixing on a plate shaker. The plate was incubated under tinfoil at 37 °C for 30 minutes. Finally, after cooling to room temperature, the absorbance was determined at 562 nm (Multiscan SkyHigh, Thermo Fisher Scientific).

1.6. Activity assay of immobilized CFE in aqueous media

This standard assay was performed in KPi buffer (50 mM, pH 6.0) using ferulic acid as substrate at an initial concentration of 10 mM, ensuring zero-order kinetics. For the former, 950 µL KPi buffer was mixed with 50 µL FA stock (200 mM in DMSO) and heated to 30°C. To initiate the reaction, 10 mg of carrier beads were added, and the mixture was subjected to 30°C and 1200 rpm. Samples of each 40 µL were quenched and diluted by adding 960 µL water/acetonitrile (1/1, v/v) and subjected to HPLC analysis. Standard sampling times were 0, 2, 5, 10, and 15 min. All derived kinetic data are based on substrate depletion.

1.7. Reaction intensification in the rotating bed reactor

The configuration of the rotating bed reactor (RBR) S2 was employed in conjunction with the V2 reaction vessel (SpinChem AB, Sweden). Immobilized PAD N31 was positioned within the mesh-lined basket of the reactor, which was subsequently filled with a solution containing the substrate. The quantity of immobilized enzyme used was equivalent to 573 U, detailed reaction conditions are stated in Table 2. CO_2 was provided by a sparger at the bottom of the reactor. Concurrently, the rotational speed was increased to mitigate mass-transfer limitations. Samples of 500 µL were collected, and substrate concentration was quantified via HPLC. To perform the basket exchange, the reaction was temporarily halted, and the RBR was withdrawn from the reaction medium. To ensure maximum recovery of the solvent and product and to minimize carry-over, the RBR was spun in the headspace for approximately two minutes, utilizing centrifugal drainage to clear the basket. Following this, the spent biocatalyst was removed, and the basket was cleaned. A fresh batch of immobilized enzyme was then weighed into the basket before reinstallation and the resumption of the reaction.

1.8. Miscibility studies

For the miscibility experiments of Bet-Gly (1:2) 80 vol.% with 20 vol.% 50 mM KPi (pH 6) and different organic solvents (Me-THF, CPME, MTBE, EtOAc, heptane). Therefore, 500 µl DES and 500 µL of the respective organic solvent were vortexed for 15 min, followed by an appropriate waiting period to allow the two phases to separate. Afterwards, 0.1 mL of the organic phase was sampled and measured by NMR (Bruker ULTRASHIELD 400 MHz. $CDCl_3$ was calibrated to 7.26 (1H) and 77.36 (13C)).

1.9. Extraction studies and determination of log K values

The log K was determined by solving 40 mM ferulic acid and 40 mM of 4-vinyl guaiacol in 80 vol.% Bet-Gly (1:2) with 20 vol.% KPi (50 mM, pH 6). 500 µl of DES and 500 µl of the respective organic solvent. The solution was vortexed for 15 min and left standing for phase

separation afterwards. 40 µl of each phase was sampled and diluted with 960 µl acetonitrile / water and subsequently subjected to HPLC analysis. The partition coefficient (log K) was determined with equation 1.

$$\log(K) = \frac{c(FA)_{org}}{c(FA)_{DES}} \tag{1}$$

1.10. Configuration of closed and open systems within smaller-scale reactions

The reaction was performed in 80 vol.% Bet-Gly (1:2) with ferulic acid as the substrate at an initial concentration of 300 mM, heated to 50°C, and a final volume of 10 mL in a 20 mL reaction vessel. To start the reaction, 14 U of carrier beads were added and the mixture subjected to 50°C and 540 rpm. Samples of each 20 µL were quenched and diluted by the addition of 980 µL water/acetonitrile (1/1, v/v), further diluted 1-to-25, and subjected to HPLC analysis. Standard sampling times were 0, 5, 15, 30, 60, and 120 min. All derived kinetic data are based on substrate depletion. To mimic a closed system, the vial was sealed with a screw cap and was opened only for sampling.

1.11. CO_2 supplementation protocol

The reaction was performed in 80 vol.% Bet-Gly (1:2) with ferulic acid as the substrate at an initial concentration of 300 mM, and the reaction mixture was heated to 50 °C in a silicone oil bath, with a final volume of 10 mL, in a 20 mL reaction vessel fitted with a silicone screw cap. To initiate the reaction, 14 U of carrier beads were added, and the mixture was incubated at 50°C and 540 rpm. CO_2 was introduced into the system via a syringe for 10, 30, and 60 min. Samples of each 20 µL were quenched and diluted by adding 980 µL water/acetonitrile (1/1, v/v), further diluted 1-to-25, and subjected to HPLC analysis. Standard sampling times were 0, 5, 15, 30, 60, and 120 min. All derived kinetic data are based on substrate depletion.

1.12. Smaller-scale reaction with higher buffer capacity or higher buffer content

This assay was performed in 80 vol.% Bet-Gly (1:2) and 20 vol.% KPi buffer (1 M, pH 6.0) using ferulic acid as substrate at an initial concentration of 300 mM. For higher buffer content, the reaction was carried out in 75 vol.% Bet-Gly (1:2), and 25 vol.% KPi buffer (50 mM, pH 6.0). To initiate the reaction, 14 U of immobilized enzyme was added, and the mixture was incubated at 50 °C and 540 rpm. Samples of each 20 µL were quenched and diluted by addition to 980 µL water/acetonitrile (1/1, v/v), further diluted 1-to-25, and subjected to HPLC analysis. Standard sampling times were 0, 5, 15, 30, 60, and 120 min. All derived kinetic data are based on substrate depletion.

2. Additional Information

2.1. Reactor cultivation of PAD N31

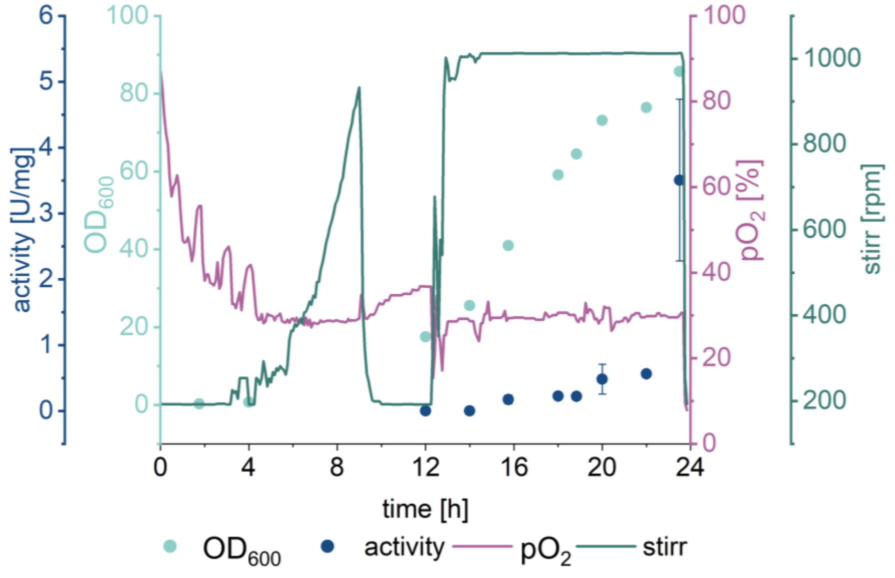

Figure S1. Process parameters of the developed bioprocess for PAD N31. Depicted are OD_{600} [-], activity [U/mg], pO_2 [%], and stirring rate [rpm] as functions of process time [h].

Upscaling to a bioreactor was performed using a 2 L Biostat A+ (Sartorius). With the selected parameters (as stated in chapter 1.1), a cell wet weight (CWW) of 114.44 g and a lyophilized CFE with an activity of 4 U/mg were achieved.

2.2. SDS-PAGE analysis of the CFE of PAD N31

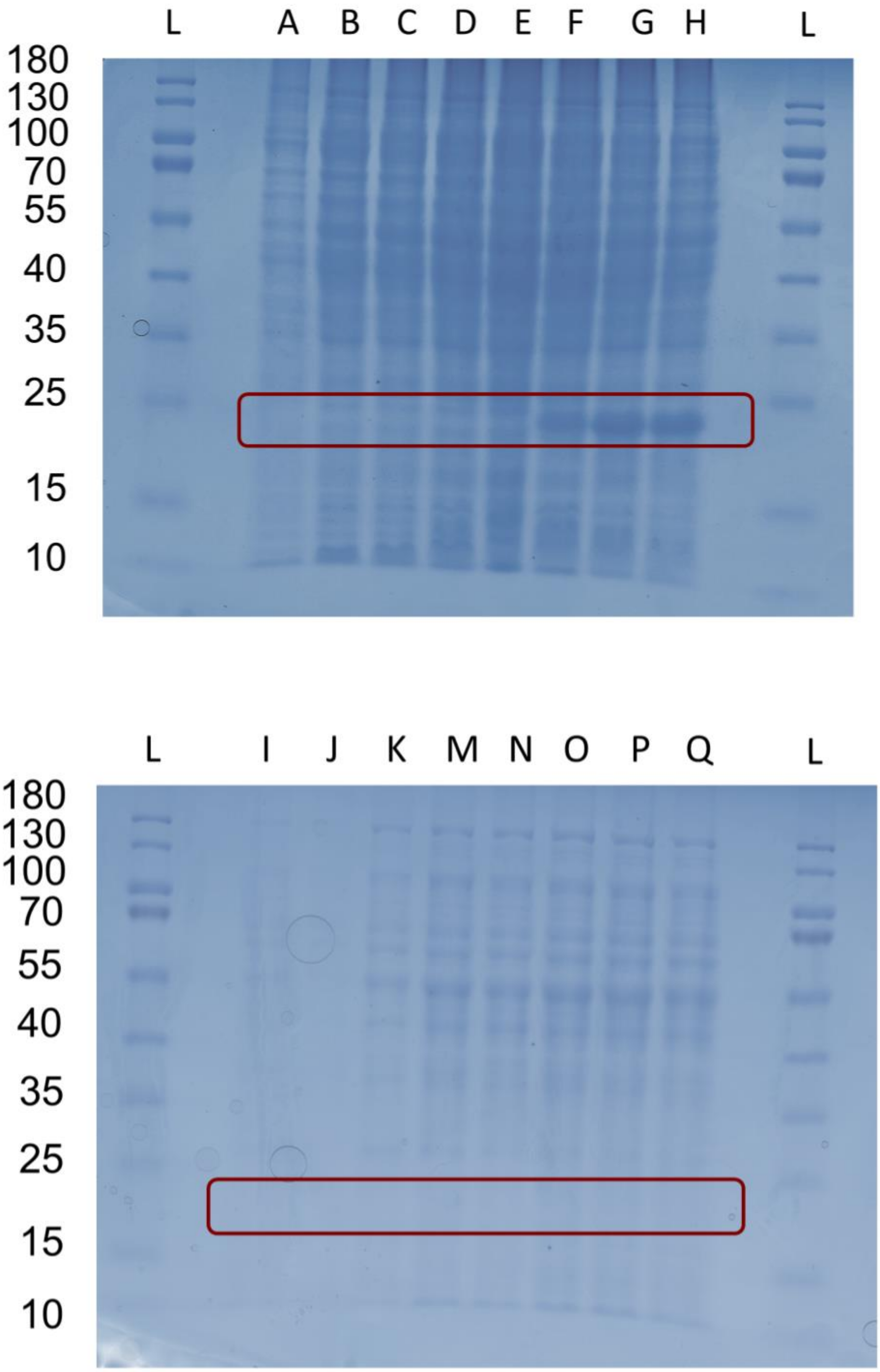

Figure S2. SDS-PAGE (12%) analysis of PAD N31. Left: CFE PAD N31 (A – 0 h, B – 4 h, C – 12 h, D – 15.75 h, E – 18 h, F – 18.8 h, G – 20 h, H – 22 h); right: debris of PAD N31 (I – 0 h, J – 4 h, K – 12 h, M – 15.75 h, N – 18 h, O – 18.8 h, P – 20 h, Q – 22 h). CFE: cell-free extract, L = PageRuler Prestained Protein Ladder (Thermo Scientific). The M.W. of PAD N31 monomer is around 21 kDa (highlighted in red box).

2.3. Exemplary HPLC chromatograms and calibration of ferulic acid in different DESs

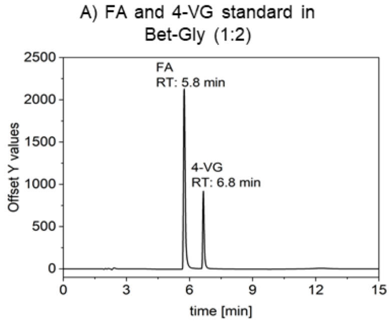

Figure S3. HPLC chromatogram of the reaction components FA (RT: 5.8 min) and 4-VG (RT: 6.8 min) in 80 vol.% Bet-Gly (1:2).

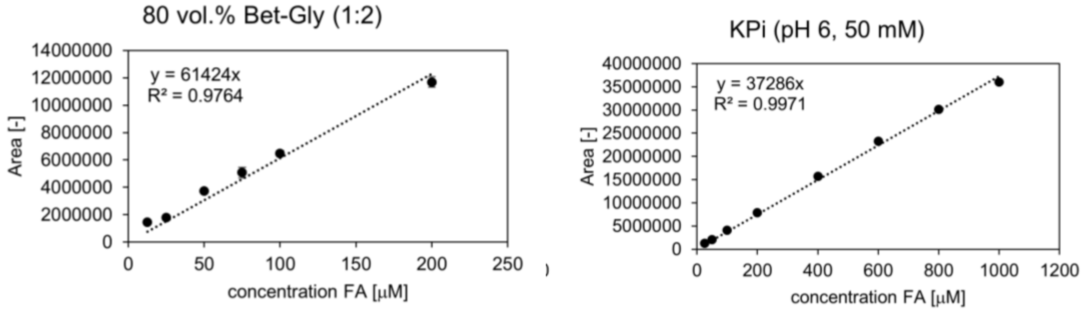

Figure S4. Calibration of ferulic acid in different DESs. Triplicates in water/acetonitrile 1/1; 10 µL injection volume; $\lambda_{detection}$ = 310 nm.

2.4. Leaching experiments of PAD N31 from the carrier in DES

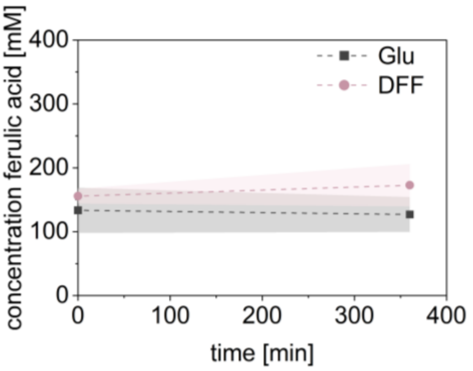

Figure S5. Leaching of PAD N31 (Seplife EMC7120M) was assessed in 1 mL of deep eutectic solvent (DES) containing 20 vol.% buffer and 40 mM ferulic acid (FA). Immobilized enzyme (120 mg) was added to initiate the reaction. Assays were conducted at 50 °C and 540 rpm in a thermoshaker for 3 h. At the end of the incubation, 100 µL of the reaction mixture was withdrawn. From this aliquot, 40 µL subsamples were taken at 0 and 360 min, quenched with 960 µL of water/acetonitrile (1:1, v/v), and subsequently diluted 1:50. Samples were analyzed by HPLC–UV.

2.5. 10 mL vessel reaction with 20% more buffer

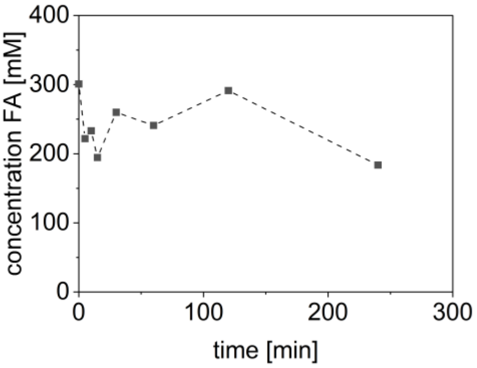

Figure S6. Progressive curve of the decarboxylation of 300 mM ferulic acid in a 10 mL vessel. Reaction conditions: 10 mL reaction volume containing 300 mM ferulic acid and 14.1 U immobilized PAD N31 at 50°C at 540 rpm in Bet-Gly (1:2) 60 vol.% and 40 vol.% KPi (50 mM, pH 6). Lines connecting experimental data are for illustrative purposes only.

2.6. pH measurement after reaction

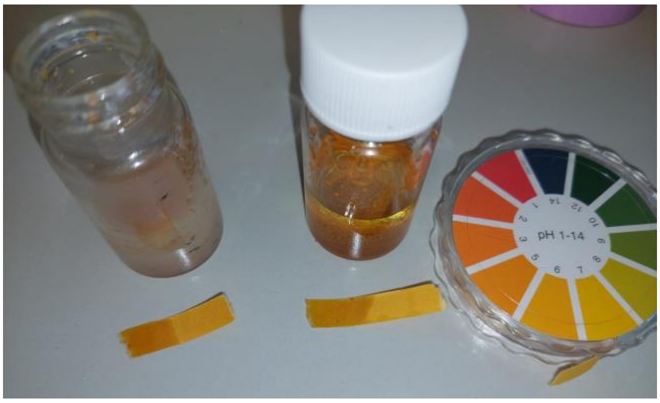

Figure S7. pH measurement using a pH strip after 24 h. Reaction conditions: Decarboxylation of 300 mM ferulic acid in 80 vol.% Bet-Gly and 20 vol.% KPi (50 mM, pH 6) with 14 U immobilized enzyme at 50 °C and 540 rpm in a closed and open system. The pH was measured using pH strips, as the highly viscous suspension is not suitable for reliable measurement with a conventional pH electrode.

2.7. RBR reaction with continuous CO_2 addition

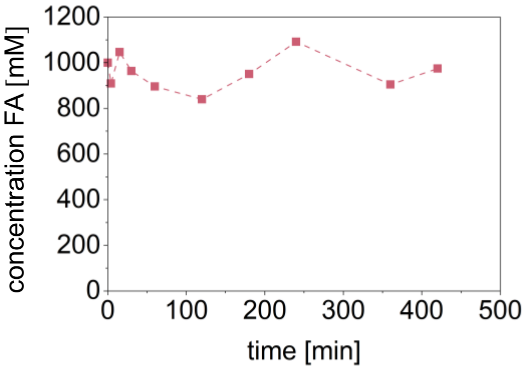

Figure S8. Progressive curve of the decarboxylation of 1 M ferulic acid (FA) in an RBR. Reaction conditions: 120 mL reaction volume containing 1 M ferulic acid and 573 U immobilized PAD N31 at 50°C at 600 rpm. Lines connecting experimental data are for illustrative purposes only. RBR = rotating bed reactor.

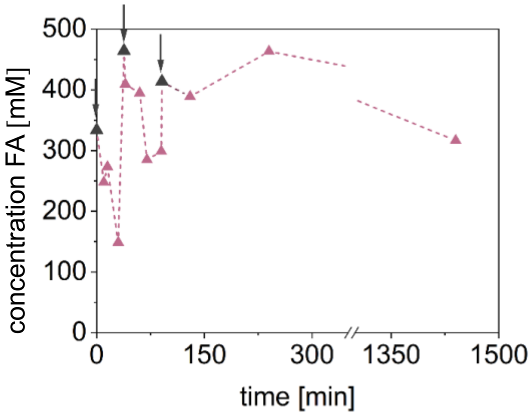

Figure S9. Fed-batch reaction in RBR. Reaction conditions: 120 mL reaction volume containing 890 mM ferulic acid (FA) and 573 U immobilized PAD N31 at 50°C and 600 rpm. The substrate was added in 3 different batches (0 min: 333 mM, 60 min: 333 mM, and 120 min: 249 mM), as indicated by the black arrows. Lines connecting experimental data are for illustrative purposes only. RBR = rotating bed reactor.

2.8. Sequential enzyme addition in the RBR

Subsequently, the total enzyme loading was increased by 28%, from 573 U to 801 U, and the enzyme was introduced via a sequential addition protocol. Enzyme beads were added at 0 min, 120 min, and 240 min (indicated by arrows in Figure 4A). During each basket exchange required for enzyme addition, a temporary increase in the measured FA concentration was observed (Figure 4A, Table 2, entry 5.0). This effect is most likely attributable not to the enzyme addition itself, but to the brief mechanical disturbance and altered mixing conditions during basket replacement. Since the reaction medium already formed a slurry, indicating operation close to the solubility limit of FA, this handling step may have temporarily changed the measurable FA concentration by redistributing suspended or locally concentrated substrate fractions.

After completion of the basket exchange and renewed homogenization, the measured FA concentration decreased again. Despite the increased overall enzyme loading, the conversion remained unchanged at ~60%, further supporting the hypothesis that FA availability in the DES system depends strongly on the applied process conditions. A comparison with the control experiment, which was started with the full enzyme loading of 573 U at t = 0 min (Figure 4B, Table 2, entry 4.3), showed a comparable kinetic profile and a slightly higher final conversion of 59%. As expected, the control reaction reached its final conversion faster (~120 min) than the sequential addition experiment, which started with only 286.5 U and reached its plateau after ~240 min.

2.9. NMR spectra of Bet-Gly

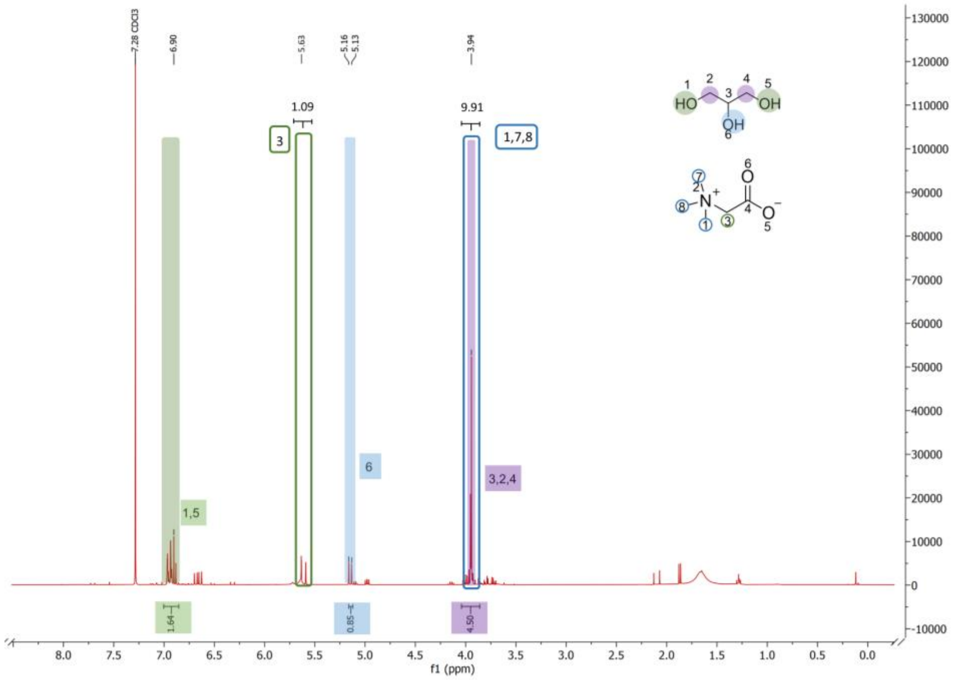

^{1}H NMR ($CDCl_3$, 400 MHz), Gly: δ 7.00 – 6.85 (m, 3H), 5.15 (d, 1H, *J* = 11.9 Hz), 3.94 (s, 5H). Bet: δ 5.63 (dd, 2H, *J* = 17.5, 0.9 Hz), 3.94 (s, 9 H).

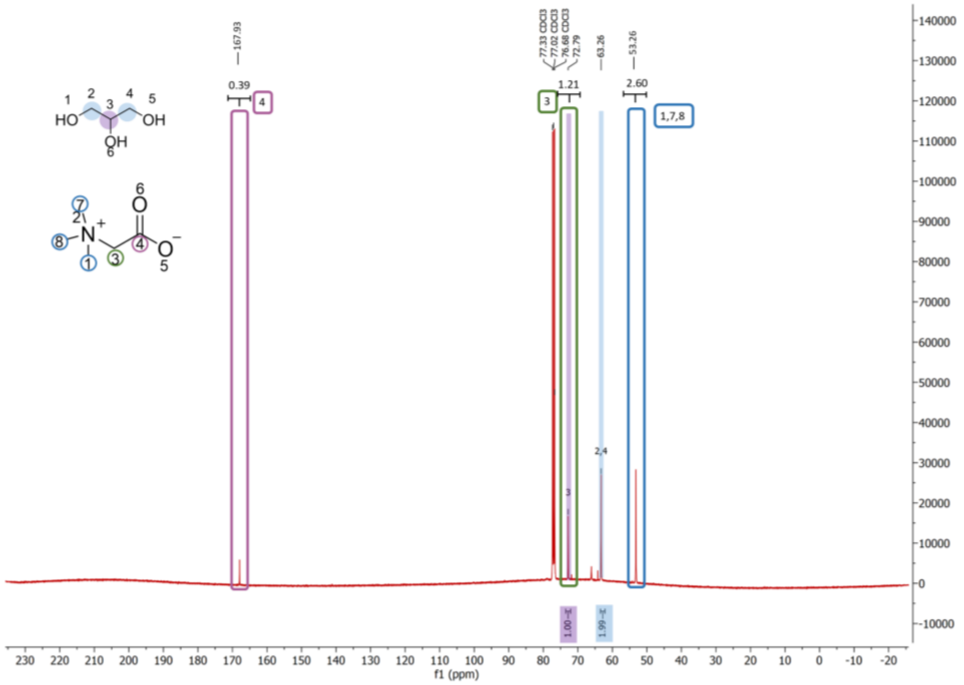

^{13}C NMR ($CDCl_3$, 101 MHz), Gly: δ 72.79 (C-3), 63.26 (C-2, C-4). Bet: δ 167.93 (C-4), 72.79 (C-3), 53.16 (C-1, C-7, C-8).

Figure S10. ^{1}H and ^{13}C NMR spectra of Bet-Gly (1:2) in $CDCl_3$.

2.10. NMR spectra of extraction solvents after contact with DES

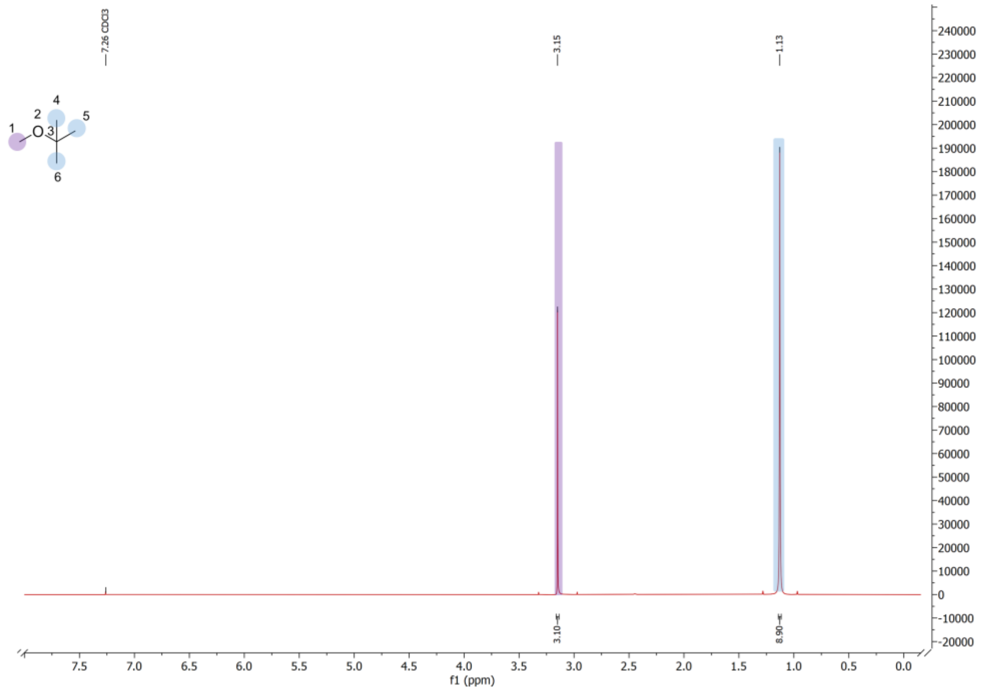

^{1}H NMR ($CDCl_3$, 400 MHz) δ 3.1 (s, 3 H), 1.13 (s, 9 H).

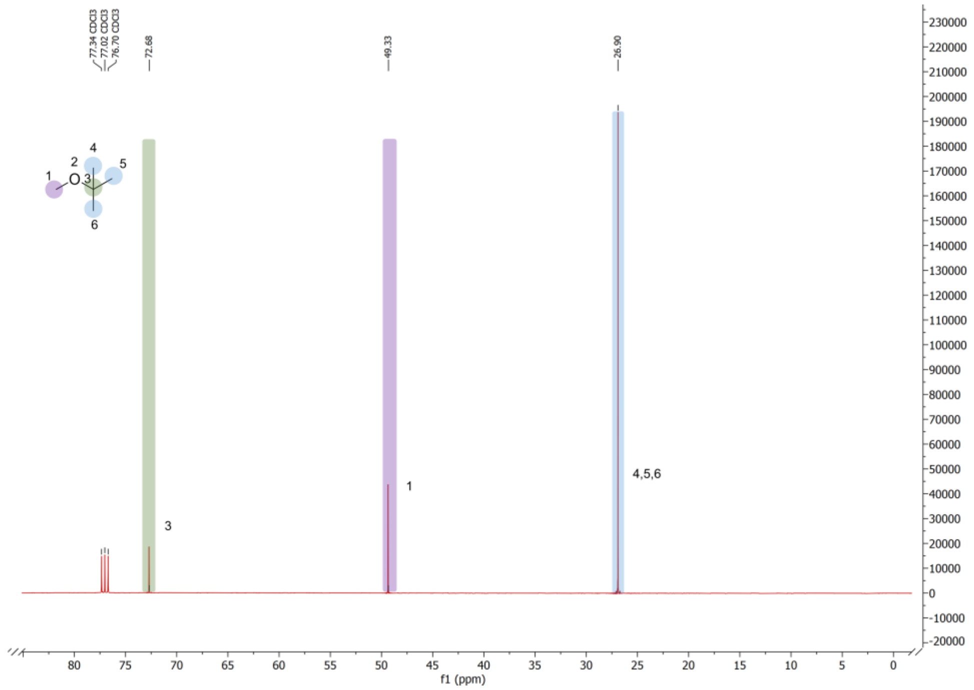

^{13}C NMR ($CDCl_3$, 101 MHz) δ 72.68, 49.33, 26.90.

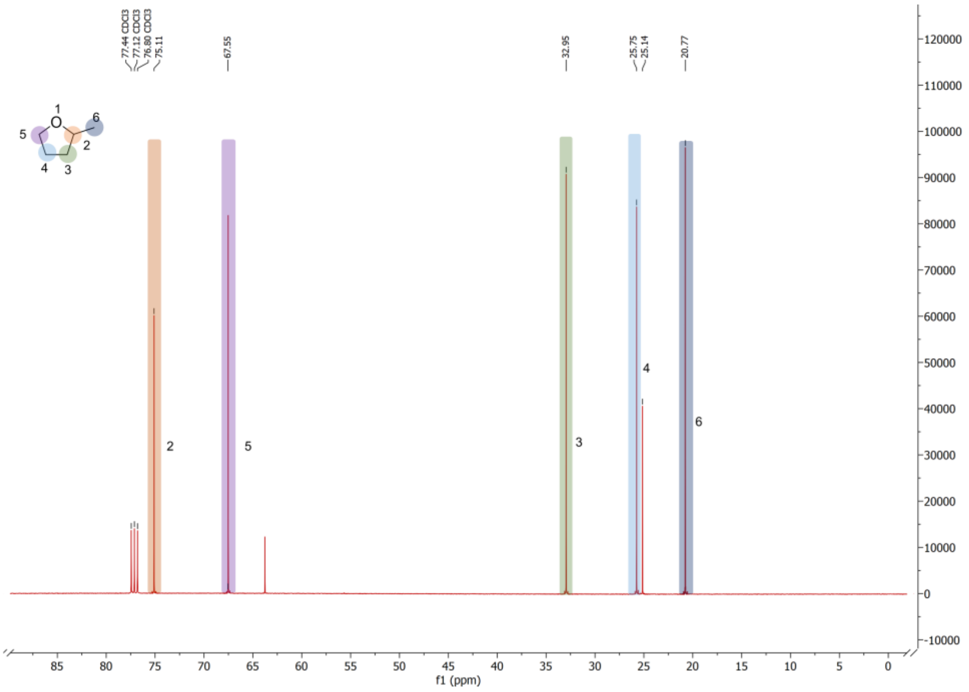

^{13}C NMR ($CDCl_3$, 101 MHz) δ 75.11, 67.55, 32.95, 25.75, 20.77.

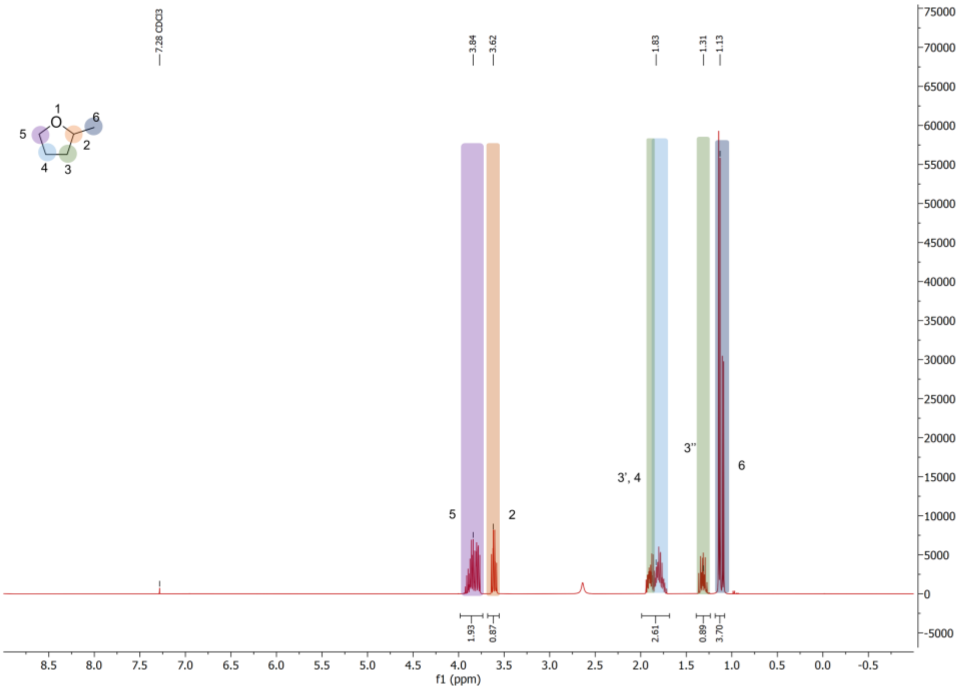

^{1}H NMR ($CDCl_3$, 400 MHz) δ 3.96 – 3.75 (m, 2H), 3.66 – 3.56 (m, 1H), 1.96 – 1.69 (m, 3H), 1.39 – 1.25 (m, 1H), 1.13 (dd, 3H, J = 16.0, 6.1 Hz).

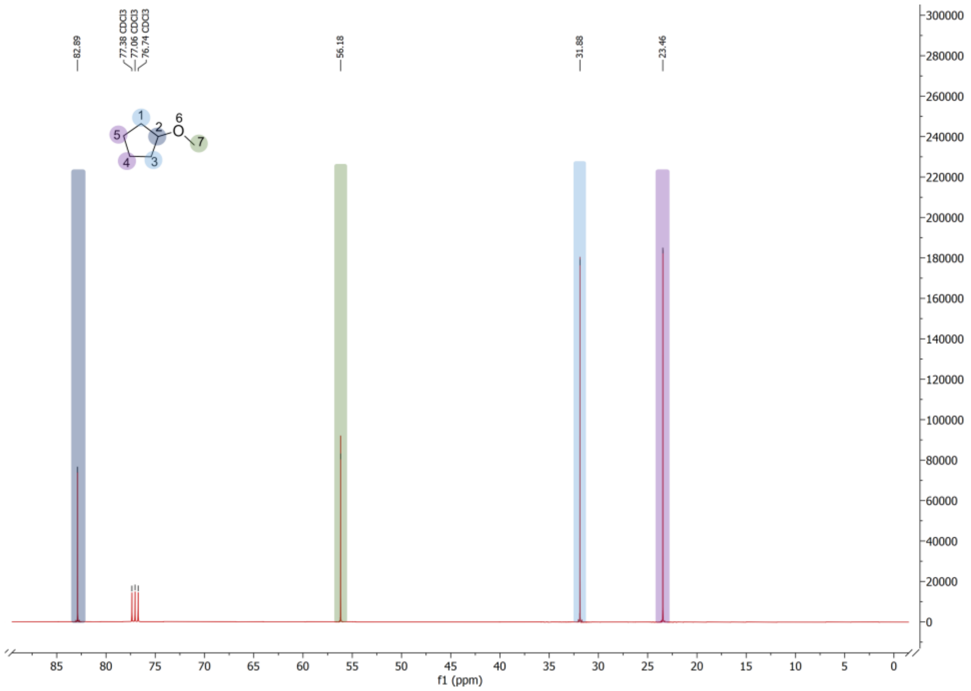

^{13}C NMR ($CDCl_3$, 101 MHz) δ 82.89, 56.18, 31.88, 23.46.

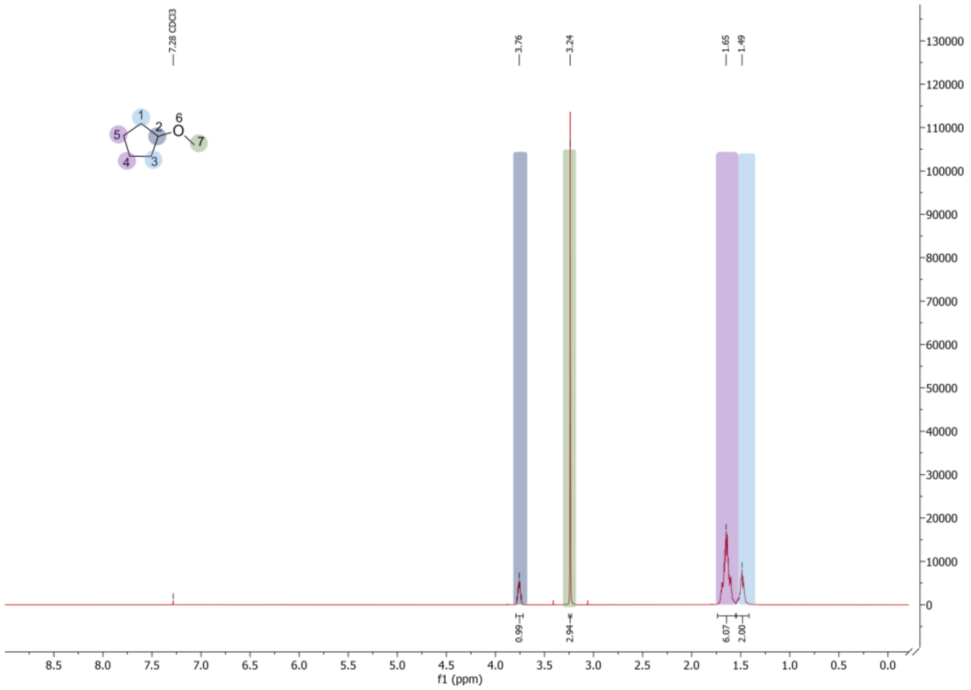

^{1}H NMR ($CDCl_3$, 400 MHz) δ 3.76 (m, 1H), 3.24 (s, 3 H) 1.68 – 1.62 (m, 6H), 1.51 – 1.41 (m, 2H).

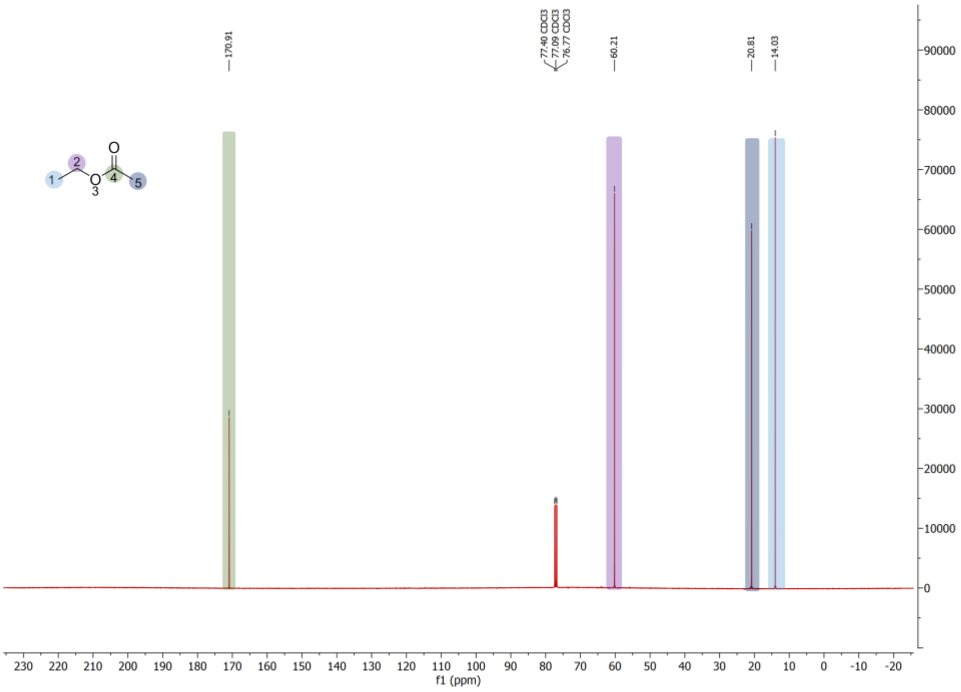

^{13}C NMR ($CDCl_3$, 101 MHz) δ 170.91, 60.21, 20.81, 14.03.

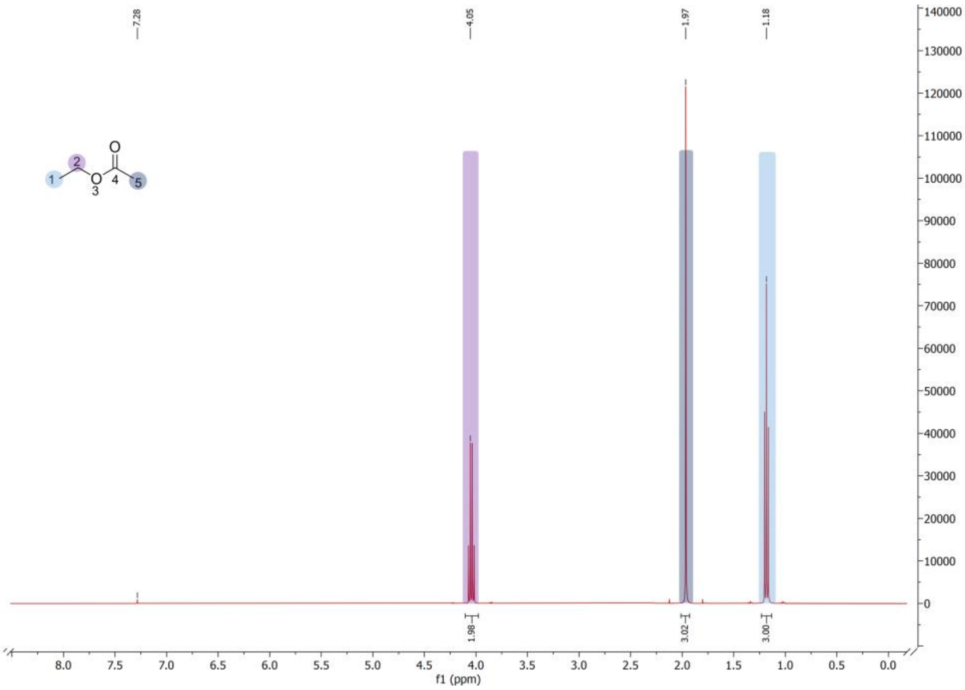

^{1}H NMR ($CDCl_3$, 400 MHz) δ 4.05 (q, 2H, J = 7.1 Hz), 1.97 (s, 3H), 1.18 (t, 3H, J = 7.2 Hz).

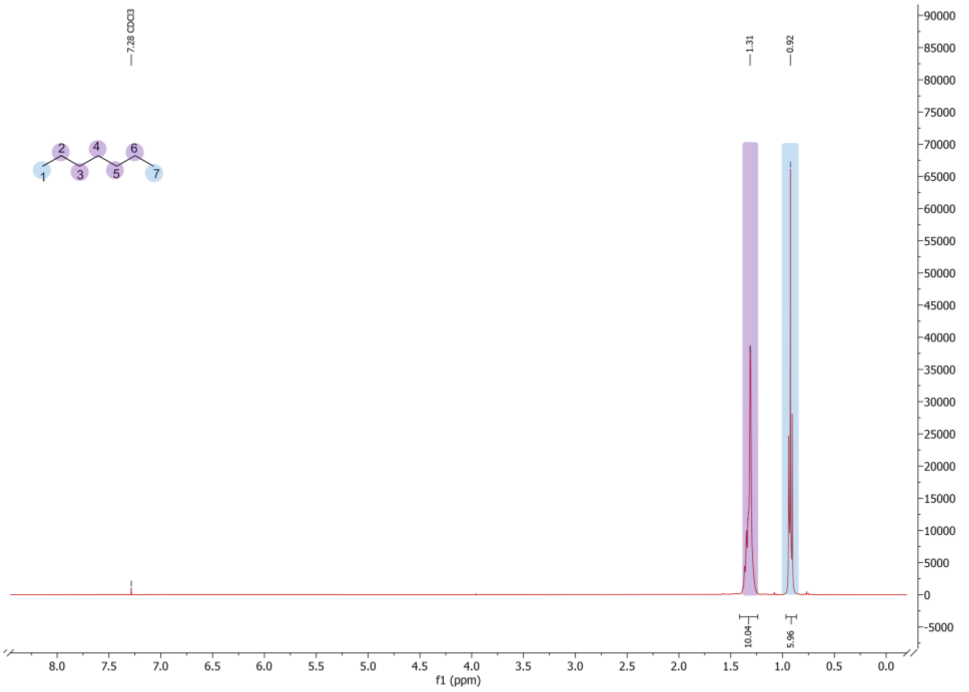

^{1}H NMR ($CDCl_3$, 400 MHz) δ 1.41 – 1.25 (m, 10H), 0.98 – 0.86 (m, 6H).

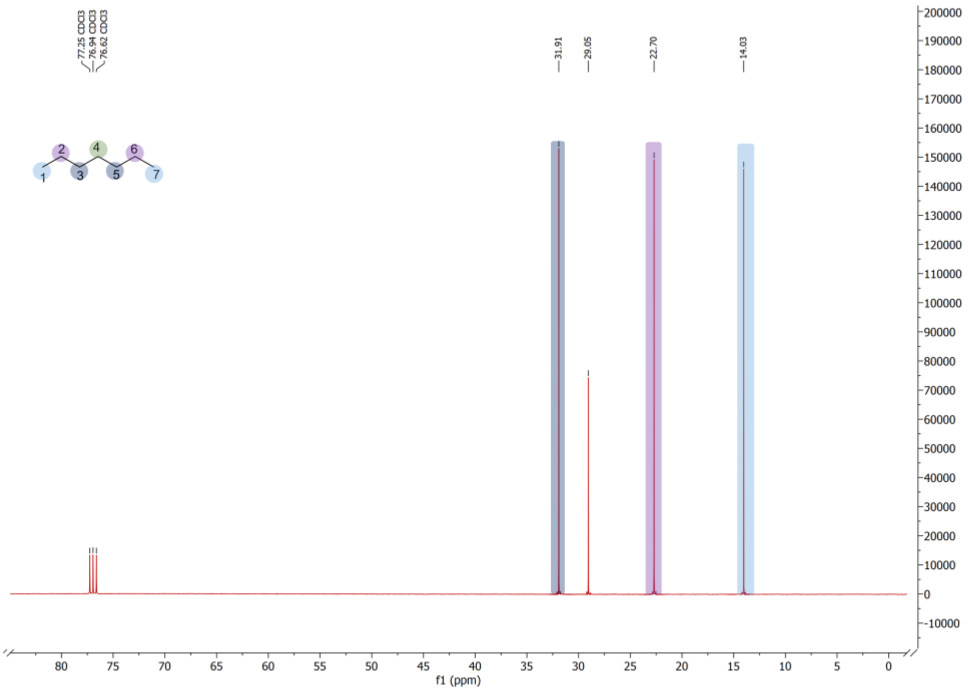

^{13}C NMR ($CDCl_3$, 101 MHz) δ 31.91, 22.7, 14.03.

Figure S11. ^{1}H and ^{13}C NMR spectra of MTBE, CPME, 2-MeTHF, EtOAc and heptane after contact with Bet-Gly (1:2) in $CDCl_3$.

2.11. HPLC spectra from the extraction experiments

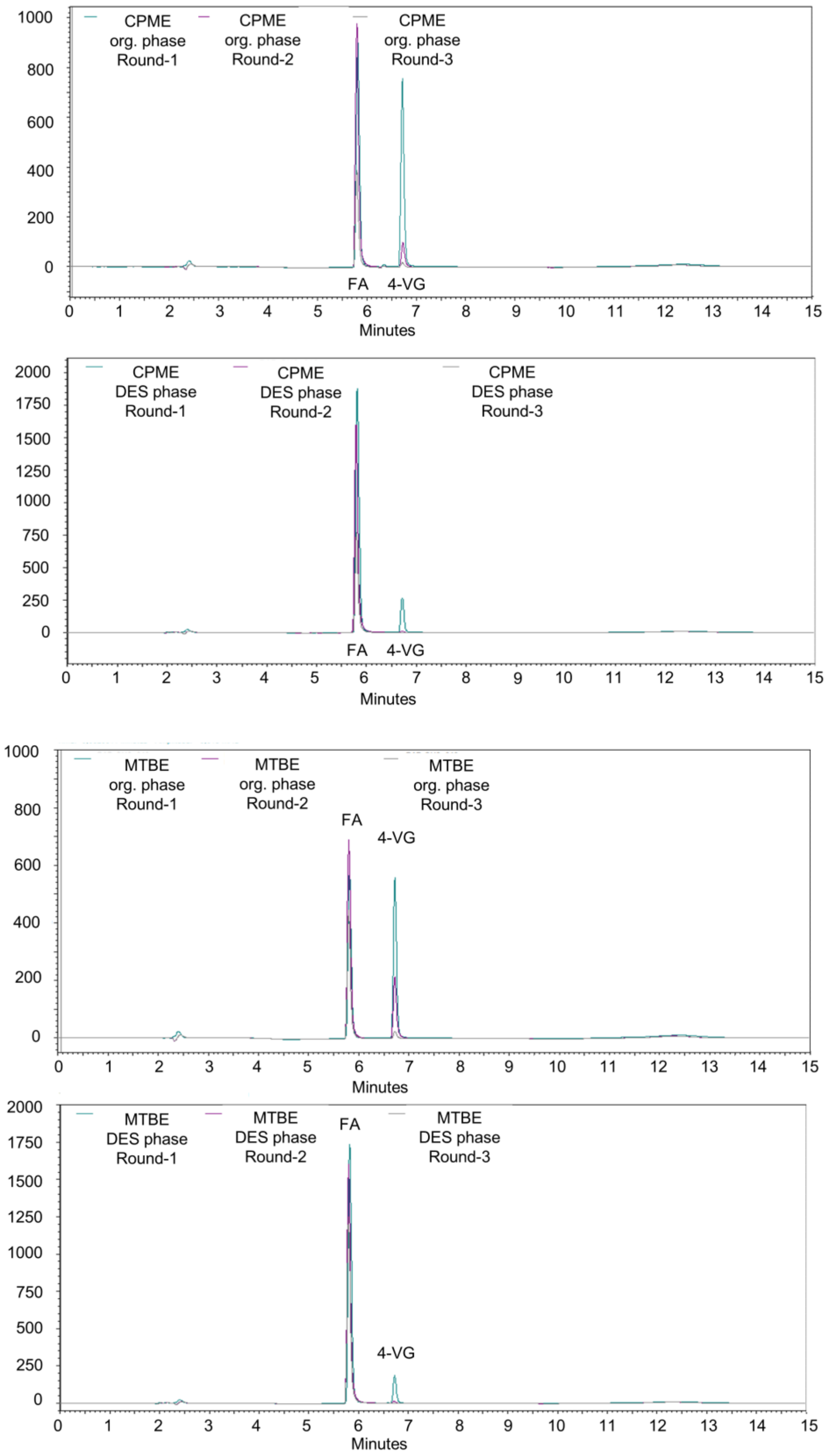

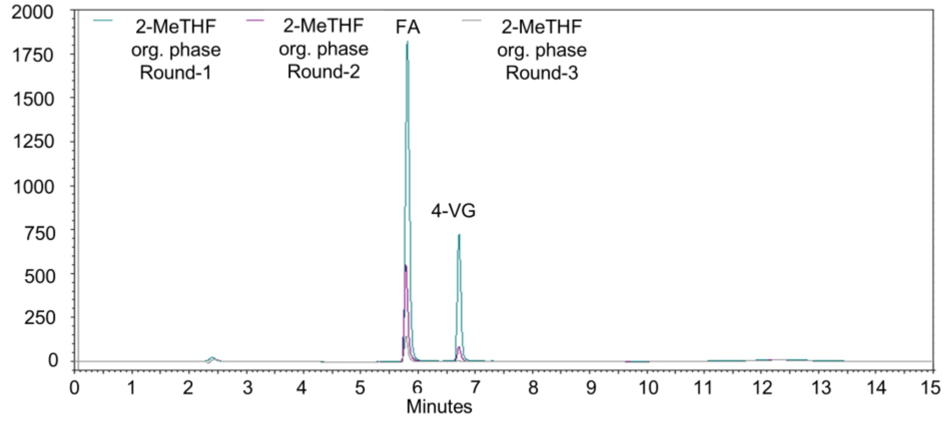
2-MeTHF org. phase Round-1
2-MeTHF org. phase Round-2
2-MeTHF org. phase Round-3
FA
4-VG
Minutes

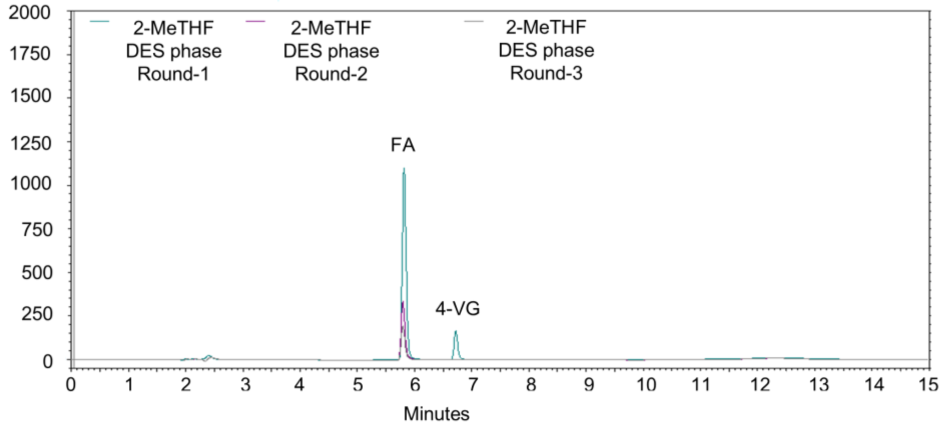
2-MeTHF DES phase Round-1
2-MeTHF DES phase Round-2
2-MeTHF DES phase Round-3
FA
4-VG
Minutes

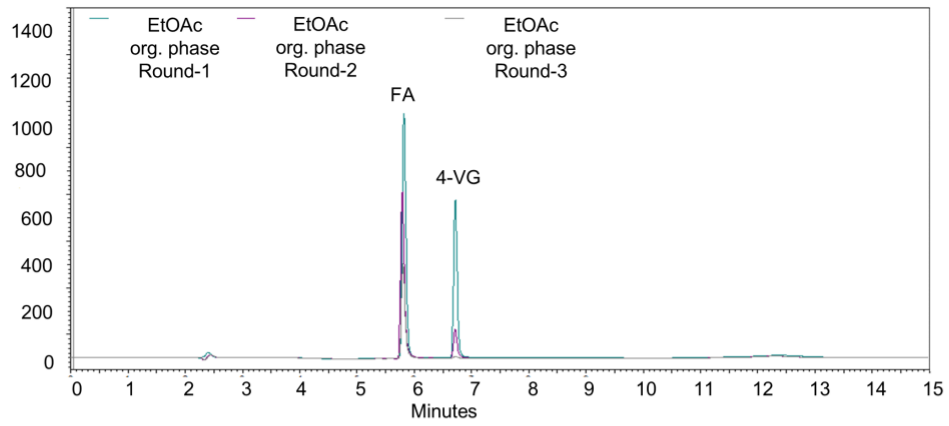
EtOAc org. phase Round-1
EtOAc org. phase Round-2
EtOAc org. phase Round-3
FA
4-VG
Minutes

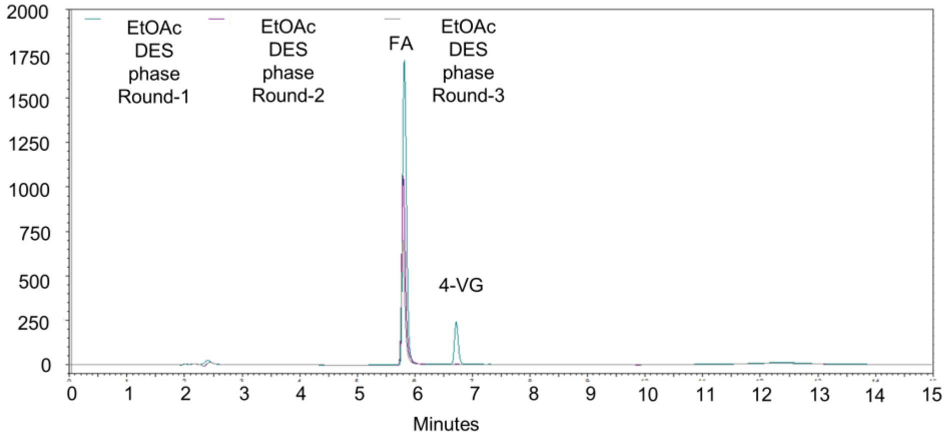
EtOAc DES phase Round-1
EtOAc DES phase Round-2
EtOAc DES phase Round-3
FA
4-VG
Minutes

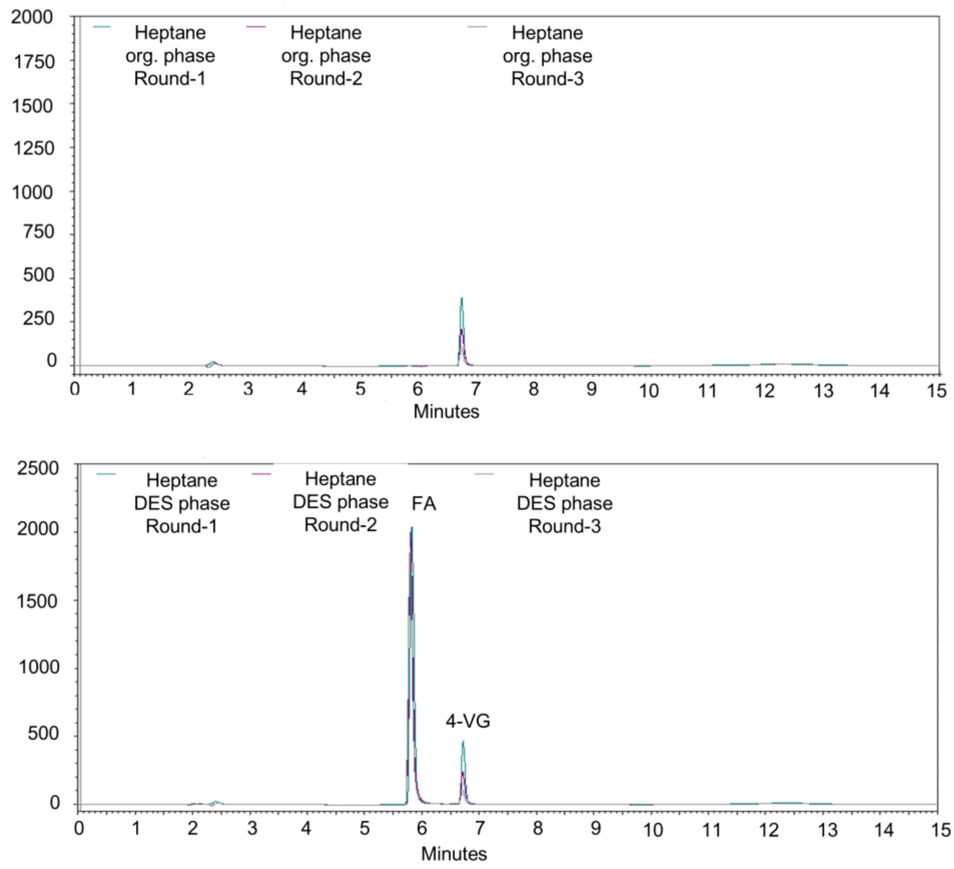

Figure S12. HPLC chromatograms of the DES phase (80 vol.% Bet-Gly and 20 vol.% KPi) as well as the organic phase (MTBE, CPME, EtOAc, heptane). MTBE = tert-methyl-butyl ether, CPME = cyclopentyl methyl ether, EtOAc = ethyl acetate, 2-MeTHF = 2-methyltetrahydrofuran.

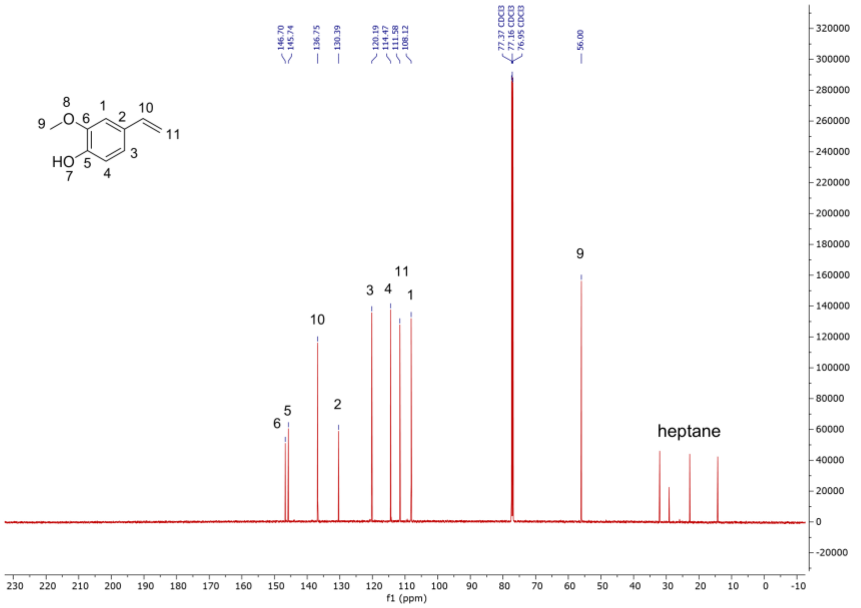

^{13}C NMR ($CDCl_3$, 101 MHz) δ 146.70, 145.74, 136.75, 120.19, 114.47, 111.58, 108.12, 56.00.

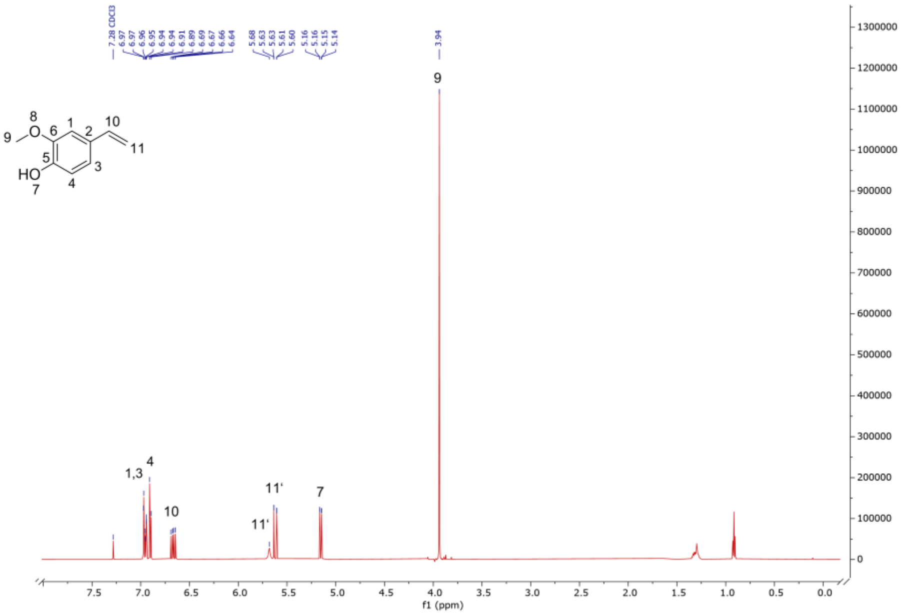

^{1}H NMR ($CDCl_3$, 600 MHz) δ 6.99 – 6.91 (m, 2H), 6.90 (d, J = 8.1 Hz, 1H), 6.67 (dd, J = 17.5, 10.8 Hz, 1H), 5.68 (s, 1H), 5.62 (dd, J = 17.5, 0.8 Hz, 1H), 5.15 (dd, J = 10.8, 0.9 Hz, 1H), 3.94 (s, 3H), 1.36 – 1.26 (m, 1H), 0.92 (t, J = 7.0 Hz, 1H).

Figure S13. ^{1}H and ^{13}C NMR spectra of 4-VG after extraction from SpinChem in $CDCl_3$.

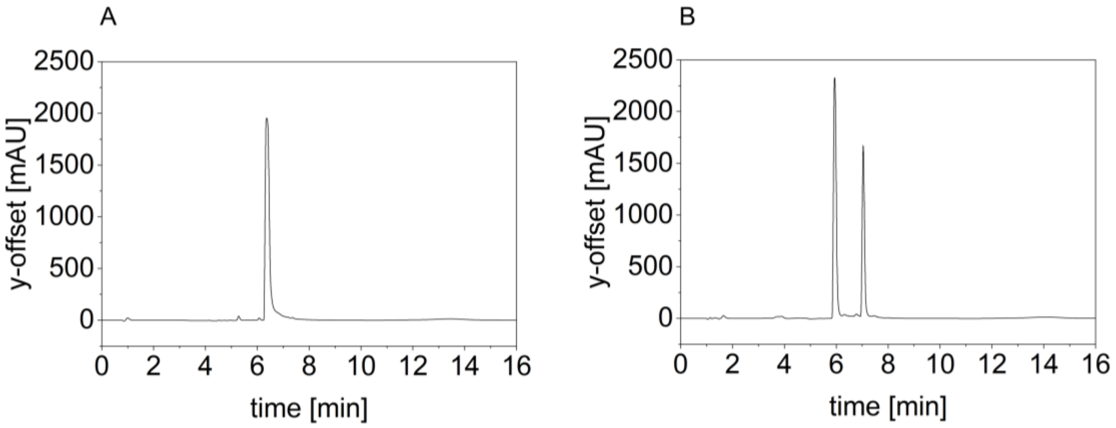

Figure S14. HPLC chromatograms of A) the heptane phase and B) DES phase after one-time extraction from SpinChem in $CDCl_3$.

3. References

(1) Li, Z.; Kessler, W.; van den Heuvel, J.; Rinas, U. Simple defined autoinduction medium for high-level recombinant protein production using T7-based Escherichia coli expression systems. *Applied microbiology and biotechnology* **2011**, *91* (4), 1203–1213. DOI: 10.1007/s00253-011-3407-z.

(2) Petermeier, P.; Bittner, J. P.; Müller, S.; Byström, E.; Kara, S. Design of a green chemoenzymatic cascade for scalable synthesis of bio-based styrene alternatives. *Green Chem.* **2022**, *24* (18), 6889–6899. DOI: 10.1039/D2GC01629J.
